# Risk of yellow fever virus importation into the United States from Brazil, outbreak years 2016–2017 and 2017–2018

**DOI:** 10.1038/s41598-019-56521-9

**Published:** 2019-12-31

**Authors:** Ilaria Dorigatti, Stephanie Morrison, Christl A. Donnelly, Tini Garske, Sarah Bowden, Ardath Grills

**Affiliations:** 1 MRC Centre for Global Infectious Disease Analysis, School of Public Health, Imperial College London, London, United Kingdom; 2 Centers for Disease Control and Prevention, Atlanta, Georgia USA; 3 Eagle Medical Services, LLC, San Antonio, Texas, USA; 4 Department of Statistics, University of Oxford, Oxford, United Kingdom

**Keywords:** Viral infection, Epidemiology

## Abstract

Southeast Brazil has experienced two large yellow fever (YF) outbreaks since 2016. While the 2016–2017 outbreak mainly affected the states of Espírito Santo and Minas Gerais, the 2017–2018 YF outbreak primarily involved the states of Minas Gerais, São Paulo, and Rio de Janeiro, the latter two of which are highly populated and popular destinations for international travelers. This analysis quantifies the risk of YF virus (YFV) infected travelers arriving in the United States via air travel from Brazil, including both incoming Brazilian travelers and returning US travelers. We assumed that US travelers were subject to the same daily risk of YF infection as Brazilian residents. During both YF outbreaks in Southeast Brazil, three international airports—Miami, New York-John F. Kennedy, and Orlando—had the highest risk of receiving a traveler infected with YFV. Most of the risk was observed among incoming Brazilian travelers. Overall, we found low risk of YFV introduction into the United States during the 2016–2017 and 2017–2018 outbreaks. Decision makers can use these results to employ the most efficient and least restrictive actions and interventions.

## Introduction

Yellow fever (YF) is an acute, vector-borne, vaccine-preventable disease caused by the YF virus (YFV) that circulates enzootically in parts of South America and sub-Saharan Africa^1–3^. YF is usually maintained in a sylvatic cycle involving mosquitoes and nonhuman primates^4^, but spillovers into the human population are frequent^5,6^. For decades, fewer than 100 human cases were reported annually in Brazil^5^. However, in 2016–2017 and 2017–2018, two large YF outbreaks caused, respectively, 784^7^ and 1,376 confirmed human cases (as of October 4, 2018)^8^ in the Southeast Region of Brazil. This region comprises the states of Espírito Santo, Minas Gerais, São Paulo, and Rio de Janeiro. Historically, yellow fever vaccination had previously only been recommended in Minas Gerais and western São Paulo states in this region; yellow fever vaccination was not recommended for eastern São Paulo state and the other two states because they were deemed not at risk of YFV transmission^3^.

Particularly concerning is that, during the 2017–2018 YF outbreak, multiple cases occurred among unvaccinated travelers who became infected with YFV while travelling in Southeast Brazil and subsequently returned to countries in South America and Europe (Argentina (3), Chile (3), Netherlands (1), France (1), Romania (1), Switzerland (1), and United Kingdom (1)) where they developed severe clinical disease leading, in some cases, to death^9^. These incidents raise concern about the risk of seeding local YFV transmission outside Brazil into countries that have competent vector mosquito populations, including the United States. As of January 2019, the United States has not reported any YFV importation from Brazil. Recent estimates suggest that the United States is the country that received the highest number of air travelers arriving in cities suitable for yellow fever transmission from YFV endemic areas worldwide^10^. Analyses of the 2016 air passenger volume data show that approximately 60% of the air travelers arriving in US cities suitable for yellow fever transmission from YFV endemic areas came from Southeast Brazil^11^. The scale of the Brazilian 2016–2017 and 2017–2018 YF outbreaks combined with the extensive interconnectedness between Southeast Brazil and the United States created concern for the importation of YFV-infected travelers^2^.

Analyses of global travel patterns have demonstrated that high connectivity is associated with increasing risks of disease emergence^10,12,13^. Mathematical models have been used in the past to quantify the risk of infection for travelers^14–16^, evaluate the probability of autochthonous transmission upon importation^17,18^ and estimate the risk of importation when incidence (the number of new cases in time) is available^19^. In a previous study, we developed a mathematical model to quantify the number of YFV importations introduced at the country level from the cumulative number of cases reported during the 2015–2016 YF outbreak in Brazil and found that the United States was at risk of YFV importation^20^. However, targeted public health prevention and preparedness planning require a more detailed analysis of where, within the United States, YFV-infected travelers are most likely to arrive. This targeting is particularly relevant because proof of yellow fever vaccination is not required to enter the United States when coming from countries at risk of YFV transmission^2^. However, the Centers for Disease Control and Prevention (CDC) recommends that travelers get yellow fever vaccination before traveling to affected areas of Brazil^21^. Additionally, the United States is currently experiencing a limited availability of yellow fever vaccine caused by an interruption in the supply of the only US licensed vaccine (YF-Vax) since July 2017 and a timeline for production of more vaccine has not been established. Although the manufacturer, Sanofi-Pasteur, gained FDA approval to import and distribute its Stamaril yellow fever vaccine in the United States under an expanded access Investigational New Drug protocol^22^, only 260 clinics across the United States have been approved to administer Stamaril (down from approximately 4,000 sites that usually distribute YF-Vax)^22^.

In this analysis, we estimate the risk of human YFV importation across all US airports with international travel from Southeast Brazil (n = 304 airports). We evaluate this risk during the 2016–2017 and 2017–2018 outbreaks in Southeast Brazil. We also explore how this risk changes with vaccination coverage.

## Results

**Figure 1:**
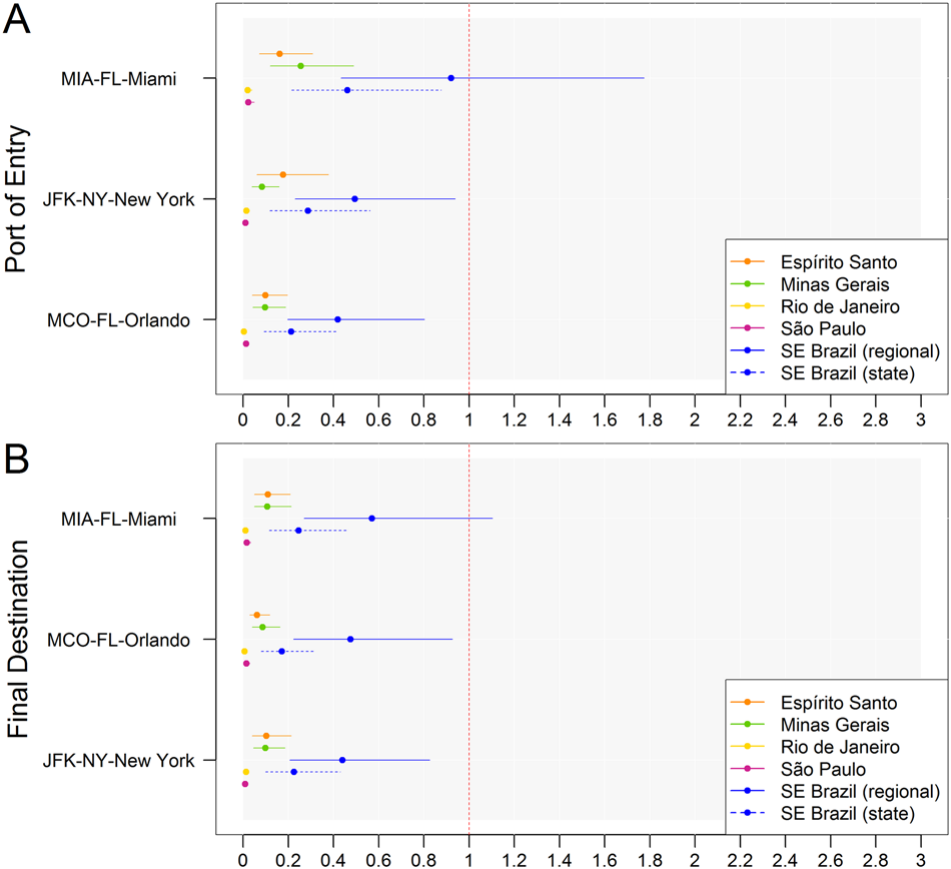
Baseline risk of YFV importation into the United States in 2016–2017. Mean and 95% confidence interval of the estimated total number of YFV importations (${I}_{S,A}^{{W}_{S}}$, comprising incoming Brazilian travelers, returning US travelers and international travelers) entering the United States at the specified ports of entry (**A**) and final destination airports (**B**) during the 2016–2017 YF outbreak. Only US airports with an upper 95% confidence limit exceeding 0.5 over all Brazilian states in Southeast Brazil (regional estimate) are shown. These estimates were obtained assuming 70% vaccination coverage of US and international travelers to Brazil. Uncertainty derives from sampling 10,000 stochastic realizations of the length of stay, incubation period, infectious period, number of asymptomatic or mild infections for each severe YFV case and vaccine efficacy from their respective distributions. SE Brazil = Southeast Brazil.

**Figure 2:**
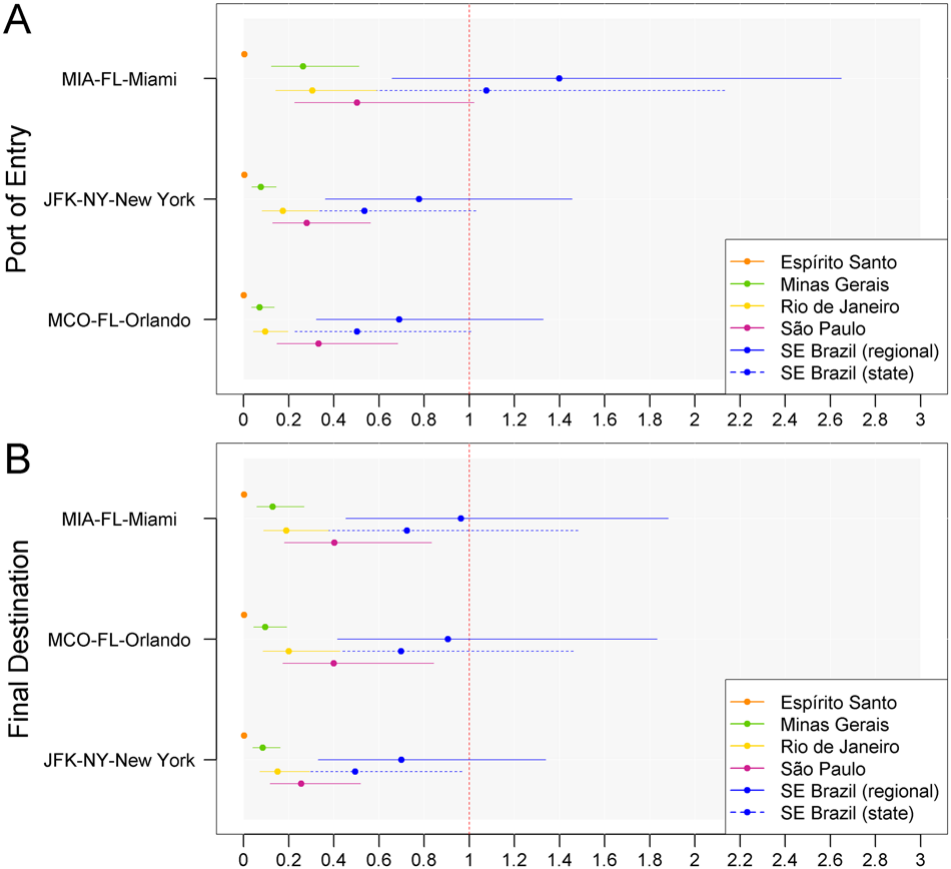
Baseline risk of YFV importation into the United States in 2017–2018. Mean and 95% confidence interval of the estimated total number of YFV importations (${I}_{S,A}^{{W}_{S}}$, comprising incoming Brazilian travelers, returning US travelers and international travelers) entering the United States at the specified ports of entry (**A**) and final destination airports (**B**) during the 2017–2018 YF outbreak. Only US airports with an upper 95% confidence limit exceeding 0.5 over all Brazilian states in Southeast Brazil (regional estimate) are shown. These estimates were obtained assuming 70% vaccination coverage of US and international travelers to Brazil. Uncertainty derives from sampling 10,000 stochastic realizations of the length of stay, incubation period, infectious period, number of asymptomatic or mild infections for each severe YFV case and vaccine efficacy from their respective distributions. SE Brazil = Southeast Brazil.

We estimated that within the United States, the Miami-MIA, New York-JFK, and Orlando-MCO international airports were at risk of receiving at least 1 YFV-infected traveler during the 2016–2017 and 2017–2018 YF outbreaks in Southeast Brazil (<1 YFV infected traveler per 100,000 travelers). This analysis included all US first points of entry (n = 90) and all US final destination airports (n = 304) with international travel volume from airports in Southeast Brazil (n = 21). Our results approximate the importation risk because we assumed that US travelers were at the same risk of YFV infection as Brazilian residents, as well as regional homogeneity in the risk of infection and in the probability of travel. Figures 1 and 2 show the mean number of YFV importations and its 95% confidence interval. Only US airports with an upper 95% confidence limit exceeding 0.5 over all Brazil’s Southeast states are shown. Specific numbers of importations among arriving Brazilian and returning US and international travelers are presented separately in the Supplementary Information (Supplementary Figs. S1–S4). They show that under the baseline assumption that 70% of US and international (non-US and non-Brazilian) travelers were vaccinated against YFV, Brazilian travelers primarily drove the risk of YFV importation at US airports during the 2016–2017 and 2017–2018 outbreaks in Southeast Brazil. Assuming state-level homogeneity of risk as opposed to regional-level homogeneity of risk of infection is generally associated with lower central estimates of the risk of YFV importation. We performed sensitivity and counterfactual analyses to test the impact of hypothetical vaccination coverage scenarios on the risk of YFV-infected travelers arriving at US airports during both YF outbreaks in 2016–2017 and 2017–2018. All regional- and state-level estimates of the total number of YFV importations (comprising Brazilian, US and international resident travelers) obtained for first US ports of entry and final destination airports in the main, sensitivity and counterfactual analyses are presented in Supplementary Tables S2–S7.

### Sensitivity analysis

#### Varying the assumed vaccination coverage of US and international travelers

In a sensitivity analysis, we tested the effect of extreme vaccination coverages of US and international travelers to Brazil on YFV importations during both outbreak years. Supplementary Figs. S7–S10 and S11–S14 show the estimated number of US and international travelers and total YFV importations (comprising Brazilian, US and international travelers) obtained under the extreme scenarios of no (i.e. 0%) and complete (i.e., 100%) vaccination coverage of US and international travelers to Brazil during the 2016–2017 and 2017–2018 YF outbreaks respectively. Under the extreme scenario of no vaccination of US and international travelers to Brazil, we found that the regional-level risk of YFV importation into US ports of entry increases to an average of 1.51 (95% CI: 0.66, 2.94) in Miami-MIA, 0.89 (95% CI: 0.37, 1.79) in New York-JFK and 0.70 (95% CI: 0.30, 1.38) in Orlando-MCO during 2016–2017 (Supplementary Fig. S5 and Table S2) and to 2.30 (95% CI: 1.00, 4.45) in Miami-MIA, 1.39 (95% CI: 0.57, 2.76) in New York-JFK, 1.06 (95% CI: 0.48, 2.01) in Orlando-MCO and 0.58 (95% CI: 0.24, 1.16) in Atlanta-ATL during 2017–2018 (Supplementary Fig. S9 and Table S6). In terms of final destination, the same risk increases to an average of 0.88 (95% CI: 0.39, 1.65) in Miami-MIA, 0.76 (95% CI: 0.31, 1.51) in New York-JFK and 0.71 (95% CI: 0.32, 1.33) in Orlando-MCO during 2016–2017 (Supplementary Fig. S5 and Table S4) and 1.47 (95% CI: 0.65, 2.80) in Miami-MIA, 1.21 (95% CI: 0.49, 2.38) in New York-JFK and 1.23 (95% CI: 0.57, 2.34) in Orlando-MCO during 2017–2018 (Supplementary Fig. S9 and Table S7). We attribute this increase to a larger number of returning US and international travelers with YFV infection (Supplementary Figs. S5 and S9) compared to the estimate obtained in the main analysis (Supplementary Figs. S2 and S4). In the absence of vaccination, the risk of importation from returning US and international (non-US and non-Brazilian) travelers becomes comparable to the risk posed by Brazilian travelers (Supplementary Figs. S1 and S3). We found that in the absence of YFV vaccination among US and international travelers visiting Brazil, the New York-JFK, Orlando-MCO and Boston-BOS international airports in 2016–2017 and the Fort Lauderdale-FLL and Atlanta-ATL international airports in 2017–2018 would also have been at risk of receiving at least 1 YFV-infected traveler (Supplementary Figs. S6 and S10).

On the other hand, even under the hypothetical scenario that all US travelers to Brazil were immunized against YFV, all airports identified as at risk of YFV importation in our main analysis (i.e., the Miami-MIA in 2016–2017, together with the New York-JFK, and Orlando-MCO international airports in 2017–2018) would still remain at risk for YFV importation (Supplementary Figs. S8 and S12). US and international travelers contribute little to the overall risk of YFV importation in presence of full yellow fever vaccination coverage of US and international travelers to Brazil (Supplementary Figs. S7 and S11).

### Counterfactual analysis

#### Varying the assumed vaccination coverage of the Brazilian populations

We conducted a counterfactual analysis to assess the impact of the vaccination campaigns conducted during the 2016–2017 and 2017–2018 outbreaks and explored the extent to which higher or lower vaccine coverage among residents of Brazil’s Southeast states would have affected the risk of YFV spread. In this analysis we estimated the counterfactual cumulative number of confirmed YF cases having assumed proportionality between the changes in the case counts and susceptibility to YFV. We then used the counterfactual number of confirmed YF cases to estimate the risk of importation due to Brazilian travelers and returning US and international (non-US and non-Brazilian) travelers (for details see Supplementary Section 4).

We found that vaccination campaigns in Brazil during the 2016–2017 YF outbreak reduced the risk of YFV importation that the United States would have faced without them (Supplementary Fig. S16). Our estimates suggest that in the absence of the 2016–2017 vaccination campaign, in addition to Miami-MIA airport identified in the main analysis, the New York-JFK, Orlando-MCO and Atlanta-ATL, international airports would also have also been at risk of YFV importation (Supplementary Fig. S16). In the absence of the 2017–2018 YF vaccination campaign, we found a significant increase in the risk of YFV importation into the Miami-MIA and Orlando-MCO international airports from the state of São Paulo (Supplementary Fig. S17), although the overall risk of YFV importation from Southeast Brazil did not substantially differ from the risk estimated in the main analysis (Fig. 1).

Our results also suggest that in the absence of substantial increases in vaccination coverage (especially in Minas Gerais) during the 2016–2017 YFV outbreak, Miami-MIA airport would have remained at risk of YFV importation (Supplementary Figs. S13–S15). This highlights the importance of outbreak containment at the source, e.g. through preventive vaccination campaigns.

The results obtained in this counterfactual analysis only account for the direct effects of vaccination. Accounting for the indirect effects of vaccination (herd immunity) would reduce the resulting estimates of the risks of YF importation.

## Discussion

This study used an established risk-modeling framework to inform potential policy decisions and actions. Results showed Miami-MIA in 2016–2017, along with New York-JFK, and Orlando-MCO airports in 2017–2018 to be at risk of receiving at least one YFV-infected traveler both as US first ports of entry and final destination airports, with the risk varying with departure state, outbreak year, vaccine coverage, and traveler type. These results are driven by the strong connectivity between Southeast Brazil and Miami-MIA, New York-JFK, and Orlando-MCO airports, which respectively acted as US first port of entry for 29% (30%), 18% (16%) and 13% (14%) of the total number of travelers entering the United States from Southeast Brazil in 2016 (2017). In terms of final destination, Miami-MIA, New York-JFK, and Orlando-MCO airports respectively received 18% (20%), 16% (15%) and 15% (17%) of the travel volume from Southeast Brazil in 2016 (2017).

Overall, we found good agreement between the regional- and state-level risk of YFV importations at US first ports of entry and final destination airports. In a sensitivity analysis, under the assumption of no vaccination of US and international travelers, we found Boston-BOS to be at risk of YFV importation in 2016–2017 as final destination airport but not as first port of entry (Supplementary Fig. S6) and Atlanta-ATL to be at risk of YFV importation as first port of entry but not as final destination airport in 2017–2018 (Supplementary Fig. S10). These results reflect the extent to which Boston-BOS mainly acts as final destination airport for travels started in Southeast Brazil through connections via other US ports of entry (of the travel volume from Southeast Brazil to Boston-BOS in 2016 and 2017, 15% entered the US at the airport and 0.4% was for transit), while Atlanta-ATL acts as a main port of entry and transit airport for the connection between Southeast Brazil and the United States (of the travel volume from Southeast Brazil to Atlanta-ATL in 2016 and 2017, 95% entered the US at the airport and 83% was for transit).

Miami-MIA, New York-JFK, and Orlando-MCO airports lie within the estimated potential range of *Aedes* mosquitoes in the United States^23^. A recent modelling study^24^ has demonstrated that high degrees of consensus and low uncertainty exist around the presence of *Ae. aegypti* in Miami and Orlando and of *Ae. albopictus* in New York, which makes these locations suitable to potential local YF transmission upon introduction. The discovery of YFV in field-collected *Ae*. *albopictus* in Southeastern Brazil in 2017, along with the demonstrated potential of YFV adaptation to *Ae*. *albopictus*to^25^ imply that autochthonous YF transmission could potentially extend to temperate regions of the United States. However, strong climatic seasonality affecting entomological traits and mosquito population dynamics^26^, along with asynchrony in the climatic patterns between the United States and Southeast Brazil, could limit the extent to which local YFV transmission upon introduction can occur.

In the main analysis we found that the average YFV importation risk in Miami-MIA was 0.92 traveler (95% CI: 0.43, 1.77) in 2016–2017 and 1.4 traveler (95% CI: 066, 2.65) in 2017–2018; in New York-JFK the average risk was 0.49 traveler (95% CI: 0.23, 0.94) in 2016–2017 and 0.78 traveler (95% CI: 0.36, 1.45) in 2017–2018; in Orlando-MCO the average risk of YFV importation was 0.42 (95% CI: 0.20, 0.80) in 2016–2017 and 0.69 traveler (95% CI: 0.32, 1.33) in 2017–2018 (Figs. 1, 2 and Supplementary Tables S2 and S6). These results are consistent with previous work by Dorigatti *et al*.^20^. During the 2016–2017 and 2017–2018 outbreaks, there were no reports of YFV-infected traveler arriving into the United States. Because of the large proportion of asymptomatic infections typically observed in YF outbreaks^27,28^, implying that a YFV-infected traveler could arrive without being detected because of the higher probability of being mild or asymptomatic than symptomatic, our estimates are in agreement with no reports of YFV importations into the United States during the 2016–2017 and 2017–2018 outbreaks. Importantly, onward transmission following introduction is a chance event. As demonstrated in^29^, the estimated levels of seeding produce high (>80%) probabilities of stochastic extinction across a wide range of transmission settings.

In the main analysis we assumed 70% vaccination coverage of US and international (non-US and non-Brazilian) travelers to Brazil and found that Brazilian travelers primarily drove the risk of importation. This result is driven by the relative proportion of Brazilian travelers (58% in 2016 and 62.5% in 2017) versus US travelers (37% in 2016 and 34% in 2017) and international (non-US and non-Brazilian) travelers (5% in 2016 and 3.5% in 2017) entering the United States and by the 70% vaccination coverage of US and international (non-US and non-Brazilian) travelers assumed in the main analysis. Sensitivity analysis on the assumed vaccination coverage of US and international (non-US and non-Brazilian) travelers shows that in the extreme hypothesis of absence of vaccination, the contribution of returning US and international (non-US and non-Brazilian) travelers to the risk of YFV importation increases to similar levels to the ones posed by Brazilian travelers (Fig. S2 vs S5 and Fig. S4 vs S9). The fact that during recent and previous outbreaks, travel-related cases occurred among returning travelers^9,21,30,31^, as opposed to residents of the source country, suggests that the actual vaccination coverage among US and international travelers may have been lower than what we have assumed in our main analysis and that international travelers may be less likely to report symptoms or seek healthcare outside their home country. Another potential reason is that returning US and international resident travelers could be at greater risk of infection than Brazilian residents, either due to increased exposure or increased susceptibility (e.g. due to lack of cross-protective immunity elicited by previous infections with other flaviviruses). A key assumption we make in this model is that the force of infection—which we define as the probability of being bitten by a YFV-infected mosquito and becoming infected with YFV—is the same for both Brazilian residents and US and international resident travelers to Brazil and differs only by the length of stay or exposure. While we recognize that there are likely heterogeneities in force of infection over time and space, as well as differences in individual behavior, it is difficult to quantify these in the absence of more detailed information. Transmission risk for vector-borne diseases is multifaceted, and fully estimating that risk would require, at minimum, information on vector presence or environmental suitability, vector abundance or density, vector biting rates, vector competence, prevalence of the pathogen in the vector population, fine-scale human density and movement, and individual behavior. Data on these variables are either unavailable or not available at a granular level to be able to properly parameterize transmission risk over time, space, and behavior.

We assumed homogeneity in the probability of traveling and the risk of infection. We assumed that US and international travelers visiting Brazil were at the same risk of YF infection as Brazilian residents due to lack of information on US and international travelers’ final destinations, activities and behavior which are all factors that could inform any differential risk of YF infection of visitors relative to the resident population. We worked with travel volume between airports, which are generally in urban areas, while most YFV transmission to humans has occurred in rural areas. Lack of data on the extent to which travelers arriving in urban areas visit rural areas during their stay may lead to overestimating the absolute or per capita risk of YFV importation. Monath *et al*. (2002) cites several factors that contribute to risk of acquiring infection, such as immunization status, geographic location, season of travel, and recreational activities^30^. However, the lack of data at the appropriate spatial and temporal scale limits our ability to differentiate between populations and their activities. Tatem *et al*. explains that such data are scattered and recommends the development of a database that combines population attributes with spatial and temporal information^32^. The granularity of the available data allowed us to explore the effect of homogenizing the risk of YF infection at the regional (Supplementary Section 1.2) versus the state (Supplementary Section 1.3) level. While increasing heterogeneity to the state level is associated with lower central estimates of the risk of YFV importation, we found that homogenizing exposure at different spatial scales generally produced comparable results. The results obtained in this analysis were obtained at the finest spatial resolution allowed by the available data. The availability of data a finer spatial resolution and the inclusion of higher degrees of heterogeneity in the model might decrease the risk of importation, but this is uncertain.

We assumed the same length of stay in Brazil for US travelers and non-US travelers entering the United States from Southeast Brazil (i.e. visiting the United States following a visit to Southeast Brazil). This assumption could have overestimated the risk of YFV importation posed by South American travelers, who on average spend 9 nights in Brazil^33^, and underestimated the risk posed by European travelers, who on average stay for 23 nights^33^ compared to the average 19 days spent in Brazil by US travelers^34,35^. We also assumed the same vaccination coverage of US and international (non-US and non-Brazilian) travelers. However, the limited travel volume of international (non-US and non-Brazilian) residents entering the United States from Southeast Brazil (5% and 3.5% of the total travel volume in 2016 and 2017, respectively) implies that non-US and non-Brazilian residents do not significantly contribute to the risk of YFV importation in the United States and that our estimates are robust to changes in the assumed length of stay and vaccination coverage of non-US and non-Brazilian travelers.

Our definition of time window (the number of days between the time of symptom onset of the first and last confirmed YFV cases) assumes no asymmetry in the temporal distribution of asymptomatic or mild YF infections compared to severe cases. Undetected or unreported transmission occurring before (or after) the detection of the first (or last) confirmed case would increase the risk of importation.

Limitations of the data used in this analysis, specifically passenger volume and vaccination coverage data, also warrant discussion. The estimates generated in this study are based on detailed aggregated data on the volume of passengers who traveled between any Brazilian airport and any US airport at a monthly temporal resolution during 2016 and 2017. While we estimated the risk of YFV importation both by first US port of entry and final destination airport, by construction these data do not capture the actual origin or destination of multicity travel itineraries. In the absence of individual-level travel information, we assumed that the origins and destinations reported in the dataset represented the actual origins and destinations of the travel itineraries.

This analysis accounted for YFV vaccination coverage estimates both among US and international travelers and in Brazil and evaluated its influence on the risk of YFV importation in the United States. For the United States, the only available vaccination coverage estimate was at the national level, which does not account for the heterogeneity in vaccination coverage likely existing across the United States. In the absence of information on the actual municipalities targeted by vaccination campaigns in Brazil in 2016–2017, we calculated the overall expected vaccination coverage in each state in Southeast Brazil. This calculation approximates the actual vaccination coverage within each state and does not capture the heterogeneity among municipalities. While a recent systematic review provides estimates on YFV vaccination coverage from 1970 to 2016^36^, it has its limitations. The population estimates used in the study do not appear to be consistent with the UN population estimates that were taken as a reference^37^ and do not account for the most recent outbreaks in Brazil.

## Conclusions

During the 2016–2017 YF outbreak in Southeast Brazil Miami-MIA, together with New York-JFK, and Orlando-MCO international airports in 2017–2018, were estimated to be at risk of YFV importation. Under the baseline scenario of 70% vaccination coverage of US and international travelers, we found that arriving YFV-infected Brazilian travelers drove the overall risk of YFV importation into the United States. This implies that outbreak containment at the source, e.g. through preventive or catch-up vaccination campaigns, is key to limit the international spread of YFV. This is particularly important as the temporary limited availability of YFV vaccine in the United States necessitates a consideration of alternative strategies, such as the targeted use of full or fractional YF vaccine doses.

This analysis informed the CDC’s Division of Global Migration and Quarantine – which strives to prevent the introduction, transmission, and spread of communicable disease using the most effective, innovative, efficient, and least restrictive actions – on the risk of YFV importation faced by US airports during the 2016–2017 and 2017–2018 YF outbreaks in Southeast Brazil. Any response needs to be commensurate with the level of risk. Because the risk of YFV importation obtained in this study yielded a relatively low risk of YFV importation and local YFV transmission following introduction, no further action among those potentially considered to be implemented (e.g. travel restriction or proof of vaccination at entry) was taken. The results presented in this study and the identification of Miami-MIA, New York-JFK, and Orlando-MCO as the main US airports at risk of YFV importation reflect the travel volume between the United States and Southeast Brazil and the scale of the YF outbreaks observed in 2016–2017 and 2017–2018. In future outbreaks, it will be necessary to reassess the risk of YFV importation and identify the new locations at risk. Similarly, to assess the risk of importation during a highly focal outbreak, like the one observed in Ilha Grande, detailed travel information would need to be included. However, models with higher complexity do not always yield greater explanatory power^18,38^.

Understanding the risk of importation through mathematical models can aid public health decision-making related to vaccination policies and other control strategies. When the ultimate objective is to prevent local YFV transmission within the United States, decision makers can consider targeted courses of action at specific ports of entry, particularly when resources are constrained.

## Methods

**Figure 3:**
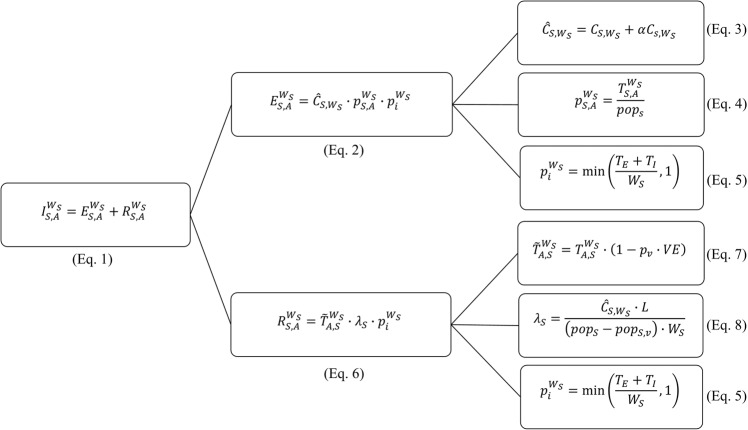
Branching tree illustrating the hierarchical nature of our risk model. The total number of importations (${I}_{S,A}^{{W}_{S}}$, left) is the sum of importations caused by incoming Brazilian travelers (${E}_{S,A}^{{W}_{S}}$, top middle) and importations caused by US and international travelers (${R}_{S,A}^{{W}_{S}}$, bottom middle). Each of these components can be further broken down into three model terms (right).

In this study, we sought to quantify the risk of YFV importation into US airports (both first ports of entry and final destination airports, separately) during the 2016–2017 and 2017–2018 YF outbreaks in Brazil’s Southeast states (i.e., Espírito Santo, Minas Gerais, Rio de Janeiro, and São Paulo). To do this, we extended the risk model framework developed by Dorigatti *et al*.^20^ to explicitly include importations from Brazilian residents (Brazilian travelers), US residents (US travelers) and international (non-Brazilian and non-US) residents (international travelers) who travelled from Southeast Brazil to the United States during the 2016–2017 and 2017–2018 YFV outbreaks in Brazil. We estimated traveler residency using OAG Aviation Worldwide Ltd.’s^11^ country of sale information (full description in Supplementary Information Section 1.1). Table 1 summarizes the notation, definitions, values and data sources used and Fig. 3 provides an illustration of the risk model adopted in the analysis.

**Table 1:** Summary of notation, data, and sources used. “ES” denotes Espírito Santo, “MG” denotes Minas Gerais, “SP” denotes São Paulo, and “RJ” denotes Rio de Janeiro. Values not listed in this table are located in the Supplementary Information. We are unable to share air passenger data because they are proprietary.

Symbol	Definition	Value	Source
${{\rm{C}}}_{{\rm{S}},{{\rm{W}}}_{{\rm{s}}}}$	Cumulative number of confirmed YFV cases reported in Brazilian state *S* during time window ${{\rm{W}}}_{{\rm{S}}}$	ES (2016–2017): 260 ES (2017–2018): 6 MG (2016–2017): 487 MG (2017–2018): 477 RJ (2016–2017): 17 RJ (2017–2018): 188 SP (2016–2017): 20 SP (2017–2018): 455	^5,7^
${{\rm{W}}}_{{\rm{S}}}$	Number of days between the time of symptom onset of the first and last confirmed YFV cases in Brazilian state *S*	ES (2016–2017): 116 days ES (2017–2018): 83 days MG (2016–2017): 122 days MG (2017–2018): 83 days RJ (2016–2017): 80 days RJ (2017–2018): 83 days SP (2016–2017): 124 days SP (2017–2018): 83 days	^5,7^
$po{p}_{S}$	Population size of Brazilian state *S*	ES: 3,973,697 MG: 20,997,560 RJ: 16,635,996 SP: 44,749,699	^39^
${p}_{V}$	Vaccination coverage of US travelers to Brazil	70%	^41^
$L$	Average length of stay of US visitors to Brazil	Empirical distribution with mean 19 days	^34,35^
$v{c}_{S}$	Average vaccination coverage of municipalities in Brazilian state *S* during the 2016–2017 YF outbreak	25.00% (<50%, Table 3 in^9^) 62.45% (50%–74.9%, Table 3 in^9^) 84.95% (75%–94.9%, Table 3 in^9^) 97.50% (≥95%, Table 3 in^9^)	^7^
${M}_{v{c}_{S}}$	Number of municipalities in Brazilian state *S* with average vaccination coverage $v{c}_{S}$ during the 2016–2017 YF outbreak	See Table 3 in^9^	^7^
$po{p}_{S,M}$	Average population size of municipalities in Brazilian state *S*	ES: 50,944.83 MG: 24,616.13 RJ: 180,826.00 SP: 69,379.38	^39^
$po{p}_{S,{V}_{2018}}$	Number of subjects vaccinated, as of April 10, 2018, during the YFV vaccination campaign started on January 25, 2018, in Brazilian state *S*	SP: 6,405,872 RJ: 1,497,840	^5^
${T}_{S,A}^{{W}_{S}}$	Number of Brazilian travelers from airports in Brazilian state *S* to US airport *A* during time window ${{\rm{W}}}_{{\rm{S}}}$ (i.e. air passengers with Brazil as country of ticket sale)	Cannot disclose (third-party data)	^11^
${T}_{A,S}^{{W}_{S}}$	Number of US and international travelers from airports in Brazilian state *S* to US airport *A* during time window ${{\rm{W}}}_{{\rm{S}}}$ (i.e. air passengers with country of ticket sale other than Brazil)	Cannot disclose (third-party data)	^11^
$\alpha$	Number of asymptomatic or mild YFV cases for each severe YFV case	Lognormal distribution with mean 7 and variance 13	^28^
${T}_{E}$	Incubation period of YFV (days)	Lognormal distribution with mean 4.6 and variance 2.7	^40^
${T}_{I}$	Infectious period of YFV (days)	Normal distribution with mean 4.5 and variance 0.6	^4,40^
$VE$	YFV vaccine efficacy	Uniform distribution between 90% and 99%	^42^

We defined importations from Brazilian travelers as Brazilian residents infected with YFV during the 2016–2017 or 2017–2018 outbreak who traveled to the United States during the incubation or infectious period (i.e. between the time of infection and the end of the period during which human blood is infectious). Similarly, we defined importations from US residents as travelers living in the United States who visited Southeast Brazil during the 2016–2017 or 2017–2018 outbreak, became infected with YFV during their stay, and returned to the United States during the incubation or infectious period. We defined importations from international (i.e. non-Brazilian and non-US) residents as international travelers who visited Southeast Brazil during the 2016–2017 or 2017–2018 outbreak, became infected with YFV during their stay, and then traveled to the United States during the incubation or infectious period. Together, these comprise the total number of YFV importations likely to arrive in the United States. In this analysis, residency was defined by the country of ticket sale. Brazilian resident travelers (hereinafter referred to as Brazilian travelers) were defined as travelers from Southeast Brazil to the United States who purchased their ticket in Brazil. Similarly, US and international resident travelers (hereinafter referred to as US and international travelers) were defined as travelers from Southeast Brazil to the United States who purchased their ticket in other countries than Brazil. To obtain a high spatial resolution of YFV importation risk, our estimates were calculated based on air travel volume from each of the four Southeast Brazilian states to all US first points of entry and final destination airports. Therefore, the total number of YFV importations (${I}_{S,A}^{{W}_{S}}$) was calculated as the sum of the number of YFV importations due to Brazilian travelers from Brazilian state *S* to US airport *A* during time window *W*_*S*_ (${E}_{S,A}^{{W}_{S}}$) and the number of YFV importations due to US and international travelers from Brazilian state *S* to US airport *A* during time window *W*_*S*_ (${R}_{S,A}^{{W}_{S}}$):

1 $${I}_{S,A}^{{W}_{S}}={E}_{S,A}^{{W}_{S}}+{R}_{S,A}^{{W}_{S}},$$

We defined the time window *W* as the number of days between the time of symptom onset of the first and last confirmed YFV cases in each Brazilian state for each outbreak (Table 1).

### Calculating importations from Brazilian travelers

The number of YFV infected Brazilian travelers arriving at US airports was calculated as:

2 $${E}_{S,A}^{{W}_{S}}={\hat{C}}_{S,{W}_{S}}\cdot {p}_{S,A}^{{W}_{S}}\cdot {p}_{i}^{{W}_{S}},$$

where ${\hat{C}}_{S,{W}_{S}}$ is the estimated total number of YFV cases in Brazilian state *S* during time window *W*_*S*_; ${p}_{S,A}^{{W}_{S}}$ is the probability of traveling from Brazilian state *S* to US airport *A* during time window *W*_*S*_; and ${p}_{i}^{{W}_{S}}$ is the probability that a YFV infected traveler is either incubating or infectious during time window *W*_*S*_. We assumed that all confirmed cases were severe and estimated the cumulative number of YFV cases in a given Brazilian state as:

3 $${\hat{C}}_{S,{W}_{s}}={C}_{S,{W}_{S}}+\alpha {C}_{S,{W}_{S}},$$

where α is the number of asymptomatic or mild YFV cases for each severe (i.e., reported) YFV case^28^. We calculated the probability of traveling from Brazilian state *S* to US airport *A* as:

4 $${p}_{S,A}^{{W}_{S}}=\frac{{T}_{S,A}^{{W}_{S}}}{po{p}_{S}},$$

where ${T}_{S,A}^{{W}_{S}}$ is the number of Brazilian resident air passengers (i.e. passengers who purchased their ticket in Brazil) traveling from airports in Brazilian state *S* to US airport *A* during time window *W*_*S*_^1,2^ and $po{p}_{S}$ is the population size of Brazilian state *S*^39^. Lastly, we calculated the probability that a YFV-infected traveler is incubating or infectious during time window *W*_*S*_ as:

5 $${p}_{i}^{{W}_{S}}=\,{\rm{\min }}(\frac{{{\rm{T}}}_{{\rm{E}}}+{{\rm{T}}}_{{\rm{I}}}}{{{\rm{W}}}_{{\rm{S}}}},1),$$

where *T*_*E*_ is the human incubation period^40^ and *T*_*I*_ is the human infectious period^4,40^ of YFV.

### Calculating importations from US and international (non-Brazilian and non-US) travelers

The number of YFV-infected US and international (non-Brazilian and non-US) travelers from Brazil was calculated as:

6 $${R}_{S,A}^{{W}_{S}}={\tilde{T}}_{A,\,S}^{{W}_{S}}\cdot {\lambda }_{S}\cdot {p}_{i}^{{W}_{S}},$$

where ${\tilde{T}}_{A,\,S}^{{W}_{S}}$ is the number of US and international (non-Brazilian and non-US) resident traveling to US airport *A* from Brazilian state *S* during time window *W*_*S*_ with no YFV vaccine-induced immunity; ${\lambda }_{S}$ is the per capita risk of YFV infection in Brazilian state *S* for US and international travelers during time window *W*_*S*_; and ${p}_{i}^{{W}_{S}}$ is the probability that a YFV infected US or international traveler flies to the United States during the incubation or infectious period (identical to Eq. 5, above). We can further decompose the number of US and international travelers without YFV vaccine-induced immunity to:

7 $${\tilde{T}}_{A,\,S}^{{W}_{S}}={T}_{A,\,S}^{{W}_{S}}\cdot (1-{p}_{V}\cdot VE),$$

where ${T}_{A,\,S}^{{W}_{S}}$ is the number of US and international resident air passengers (i.e. passengers who purchased their ticket in countries other than Brazil) entering US airport *A* from Brazilian state *S* during time window *W*_*S*_^11,25^; ${p}_{V}$ is the YFV vaccination coverage of US travelers to Brazil^41^; and $VE$ is the YFV vaccine efficacy^42^. We assumed that US and international (non-Brazilian and non-US) travelers visiting Brazil were at the same risk of exposure to YFV as the population of Brazil. Therefore, the Brazilian state-specific per capita risk of YFV infection was calculated as:

8 $${\lambda }_{S}=\frac{{\hat{C}}_{S,{W}_{S}}\cdot L}{(po{p}_{S}-po{p}_{S,V})\cdot {W}_{s}},$$

where $${\hat{C}}_{S,{W}_{S}}$$ is the estimated total number of YFV cases in Brazilian state *S* during time window *W*_*S*_^5,7^; $$L$$ is the length of stay of US travelers in Brazil^34,35^; $$po{p}_{S}$$ is the population size of Brazilian state *S*^39^; and $$po{p}_{S,V}$$ is the number of individuals with YFV vaccine-induced immunity in Brazilian state *S*. We assumed that international (non-Brazilian and non-US) travelers had the same vaccination coverage and length of stay of US travelers. The distribution of the length of stay was obtained by fitting the frequency of the number of nights US residents spent in South America reported in^35^. The term $$po{p}_{S,V}$$ for the 2016–2017 YF outbreak was given by the sum of the average population size of municipalities in Brazilian state *S*^28^ ($$po{p}_{S,M})$$, multiplied by the number of municipalities in Brazilian state *S* ($${M}_{v{c}_{S}})\,$$ with average vaccination coverage ($$v{c}_{S})$$ during the 2016–2017 YF outbreak^7^ and the YFV vaccine efficacy $$VE$$:

9 $$po{p}_{S,v}=\sum _{M}\,po{p}_{S,M}\cdot {M}_{v{c}_{S}}\cdot v{c}_{S}\cdot VE.$$

The term $po{p}_{S,V}\,$ for the 2017–2018 YF outbreak was calculated as above, having added the number of subjects vaccinated during the YFV vaccination campaign started on January 25, 2018^5^ ($po{p}_{S,{V}_{2018}}$):

10 $$po{p}_{S,v}=(\sum _{M}\,po{p}_{S,M}\cdot {M}_{v{c}_{S}}\cdot v{c}_{S}\cdot VE)+po{p}_{S,{V}_{2018}}.$$

Variability in the length of stay (*L)*, incubation periods (*T*_*E*_), infectious periods (*T*_*I*_), number of asymptomatic or mild YFV cases for each severe YFV case (α) and vaccine efficacy ($VE$) was accounted for by sampling 10,000 times from their respective distributions (Table 1). The risk estimates are directly proportional to the number of asymptomatic or mild YFV cases for each severe YFV case (α), to the probability that a YFV-infected traveler is incubating or infectious during the epidemic time window (${p}_{i}^{{W}_{S}}$) and to the vaccination coverage of US and international travelers (${p}_{V}$). The results of the analysis are reported in terms of mean and 95% confidence interval (CI) of the number of incoming Brazilian travelers (${E}_{S,A}^{{W}_{S}}$),US and international travelers (${R}_{S,A}^{{W}_{S}}$), and total number of YFV importations (${I}_{S,A}^{{W}_{S}}$) likely to arrive at individual US airports (Figs. 1 and 2).

The total number of YFV importations from Southeast Brazil into the United States was estimated in two ways: (i) by applying the framework developed for Brazilian state *S* to the cumulative number of confirmed cases, population figures, and joint travel volume from Espírito Santo, Minas Gerais, São Paulo, and Rio de Janeiro (we refer to this estimate as regional-level estimate, see Supplementary Information, Methods Section 1.2 for details) and (ii) by summing the total number of YFV importations obtained for Espírito Santo, Minas Gerais, São Paulo, and Rio de Janeiro (we refer to this estimate as state-level estimate as detailed in Supplementary Information, Methods Section 1.3).

We also performed multiple sensitivity analyses to explore the impact of uncertainty of vaccination coverage on the risk of YFV importation among both US and international residents and residents of Brazil traveling to the United States (Supplementary Information, Sensitivity Analysis section 3 and Counterfactual Analysis section 4).

## Supplementary information

Supplementary Information.

## Data Availability

All data used in this study, except the air passenger data, are summarized in Table 1. The air passenger data used in this study are proprietary and were purchased from OAG Aviation Worldwide Ltd. These data were used under United States Centers for Disease Control and Prevention license for the current study and so are not publicly available. The authors are available to share the air passenger data upon reasonable request and with the permission of OAG Aviation Worldwide Ltd.
