# Supplementary material for: Risk of yellow fever virus importation into the United States from Brazil, outbreak years 2016–2017 and 2017–2018
Source: Sci Rep. 2019 Dec 31;9:20420. doi: 10.1038/s41598-019-56521-9 (PMC6938482; doi:10.1038/s41598-019-56521-9)
Supplement: Supplementary file 1 — Supplementary Information. [file 41598_2019_56521_MOESM1_ESM.pdf]

Supplementary Information

Risk of yellow fever virus importation into the United States from Brazil, outbreak years 2016–2017 and 2017–2018

Ilaria Dorigatti[1*], Stephanie Morrison[2,3], Christl A. Donnelly[1,4], Tini Garske[1], Sarah Bowden[2,3], Ardath Grills[2]

[1] MRC Centre for Global Infectious Disease Analysis, Department of Infectious Disease Epidemiology, Imperial College London, London, United Kingdom

[2] Centers for Disease Control and Prevention, Atlanta, Georgia, USA

[3] Eagle Medical Services, LLC

[4] Department of Statistics, University of Oxford, Oxford, United Kingdom

[*]Corresponding author: email address i.dorigatti@imperial.ac.uk, phone number +44 (0)20 7594 1451

1. Methods

1.1 US Travelers and Brazilian Travelers Determination

Travel to the United States includes travel to all airports in the 50 states, the District of Columbia. In the analysis, we used airport level OAG Aviation Worldwide Ltd.'s (OAG Aviation Worldwide Ltd. OAG Traffic Analyser, Version 2.0. 2018) monthly air passenger counts from Brazil to the United States from January 2016 through December 2017.

We used the country of ticket sale as a proxy for residency, i.e. we approximated (i) the number of Brazilian residents travelling to the United States using the OAG travel volume with Brazil as point of sale, (ii) the number of US travelers returning to the United States using the OAG travel volume with United States as point of sale and (iii) the number of international (non-Brazilian and non-US) residents traveling to the United States from Brazil using the OAG travel volume with point of sale other than Brazil or the United States.

We used information provided by OAG Aviation Worldwide Ltd.'s Origin and Destination Report to determine first US port of entry (POE) and the final destination airport within the in the United States. For example, for a passenger journey started in Brazil, entered the United States at New York, JFK, and ended at Houston, IAH, the JFK airport would be the first US POE and Houston, IAH would be the final destination.

These data include inbound travel from Brazil into POEs within the United States. Travel volume numbers are modeled estimates based on ticket sales and reporting from airline carriers. Travel volume represents the aggregate number of passenger journeys, not necessarily unique individuals.

1.2 Number of YFV importations into the United States from Southeast Brazil, assuming homogeneity in the risk of YFV infection at the regional level

The total number of YFV importations entering the United States from Southeast Brazil, comprising importations from both Brazilian travelers and US travelers, was computed using the cumulative case count, demographic, and travel volume data for the states of Espírito Santo, Minas Gerais, São Paulo, and Rio de Janeiro and was given by

$$I_{\mathrm{B},A}^{W_B} = E_{\mathrm{B},A}^{W_B} + R_{\mathrm{B},A}^{W_B}.$$

Specifically, we set the temporal window W_B equal to the number of days between the first and the last confirmed YF case across all states in Southeast Brazil. We estimated the number of importations among Brazilian residents traveling from Southeast Brazil by multiplying the estimated total number of YF cases (including asymptomatic and mild YF infections) in Southeast Brazil during time window W_B by the probability of traveling from any state *S* in Southeast Brazil to US airport *A* during time window W_B and the probability that a YFV infection incubates YF or is infectious during time window W_B:

$$E_{\mathrm{B,A}}^{W_B} = \left(\sum_S \hat{C}_{S,A}^{W_S}\right) \cdot p_{\mathrm{B},A}^{W_B} \cdot p_i^{W_B} \text{, where } p_{\mathrm{B},A}^{W_B} = \frac{\sum_S T_{\mathrm{S,A}}^{W_S}}{\sum_S pop_S} \text{ and } p_i^{W_B} = \min\left(\frac{T_E + T_I}{W_B}, 1\right).$$

We estimated the overall number of YFV-infected US travelers from Southeast Brazil by multiplying the number of US and international residents traveling to Southeast Brazil during time

window W_B with no YF vaccine-induced immunity by the per capita risk of infection in Southeast Brazil during their stay and the probability of traveling to the United States during the incubation or infectious period:

$$R_{B,A}^{W_B} = \left[\left(\sum_S T_{A,S}^{W_S}\right)\cdot(1-p_v\cdot VE)\right]\cdot\lambda_B\cdot p_l^{W_B}, \quad \text{where} \quad \lambda_B = \frac{(\sum_S \hat{c}_{S,W})\cdot L}{(pop_B - pop_{B,V})\cdot W_B}, \quad pop_B = \sum_S pop_S,$$

$$pop_{B,V} = \sum_S pop_{S,V} \text{ and } p_l^{W_B} = \min\left(\frac{T_E + T_I}{W_B}, 1\right).$$

Because we assumed a homogeneous risk of YFV infection for the whole region of Southeast Brazil, we refer to the risk estimates obtained with the method described in this section as regional-level estimates.

<u>1.3 Number of YFV importations into the United States from Southeast Brazil, assuming homogeneity in the risk of YFV infection at the state level</u>

In this alternative formulation, we calculated the total number of YFV importations in the United States as the sum of the estimated Brazilian travelers and US and international travelers infected over all Southeastern states: $\tilde{I}_{B,A}^{W_B} = \sum_S E_{S,A}^{W_S} + R_{S,A}^{W_S}$. We refer to the estimates obtained with the method described in this section as state-level estimates because they assume homogeneity in the risk of YFV infection at the state level.

2. Results

Figures S1 andS2 show the estimated number of YFV importations introduced into the United States at the specified US first ports of entry and final destination airports during the 2016–2017 YF outbreaks in Southeast Brazil among Brazilian travelers and US and international travelers, respectively. Figures S3 and S4 show the same but for the 2017–2018 YF outbreaks in Southeast Brazil.

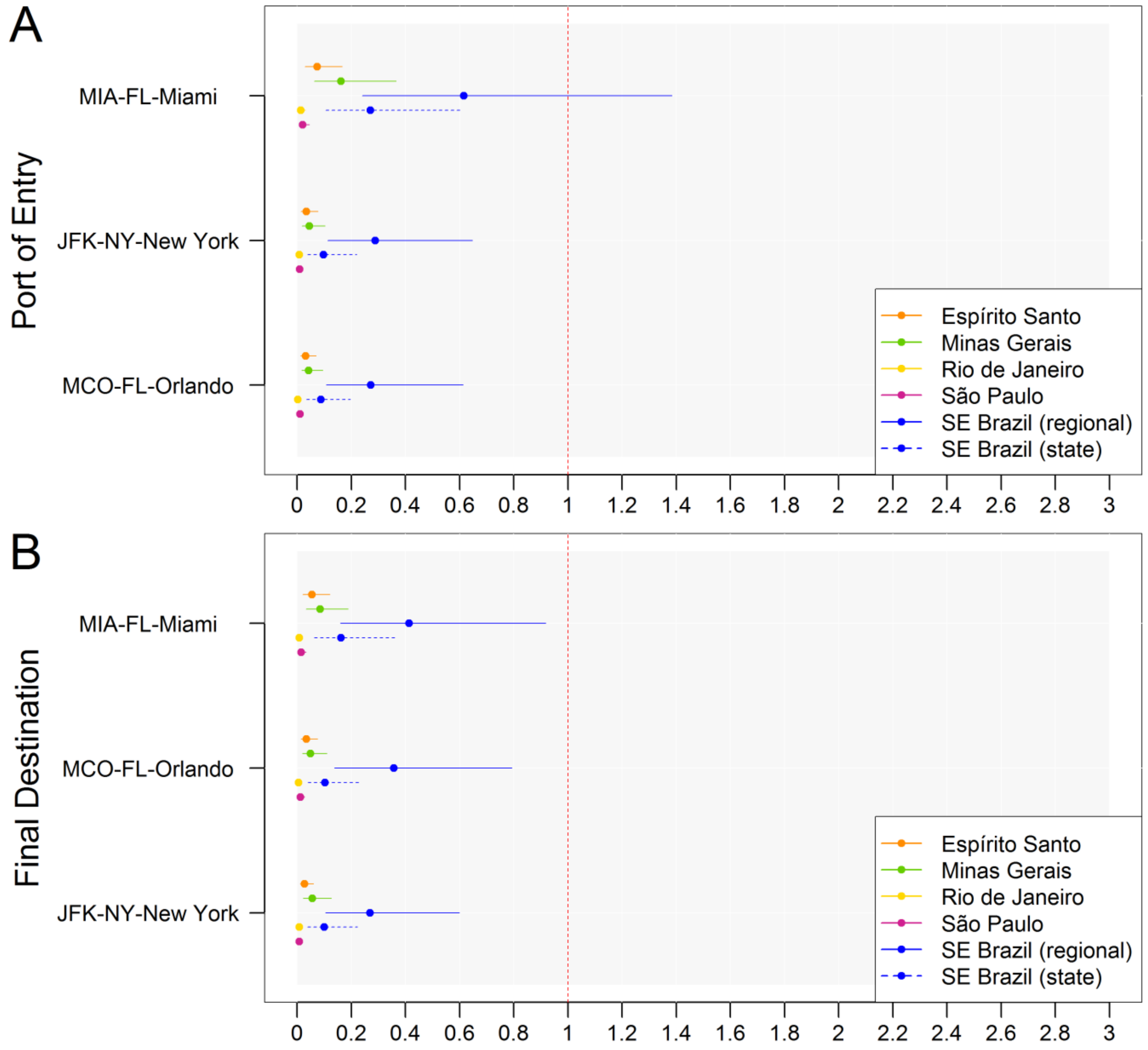

Figure S1. **Baseline risk of YFV importation from Brazilian travelers, 2016–2017 outbreak.** Mean and 95% confidence interval of the estimated number of YFV-infected Brazilian travelers ($E_{S,A}^{W_S}$) entering the United States at the specified ports of entry (A) and final destination airports (B) during the 2016–2017 YF outbreak. Only US airports with an upper 95% confidence limit of the total number of YFV importations exceeding 0.5 over Southeast Brazil (SE Brazil) are shown.

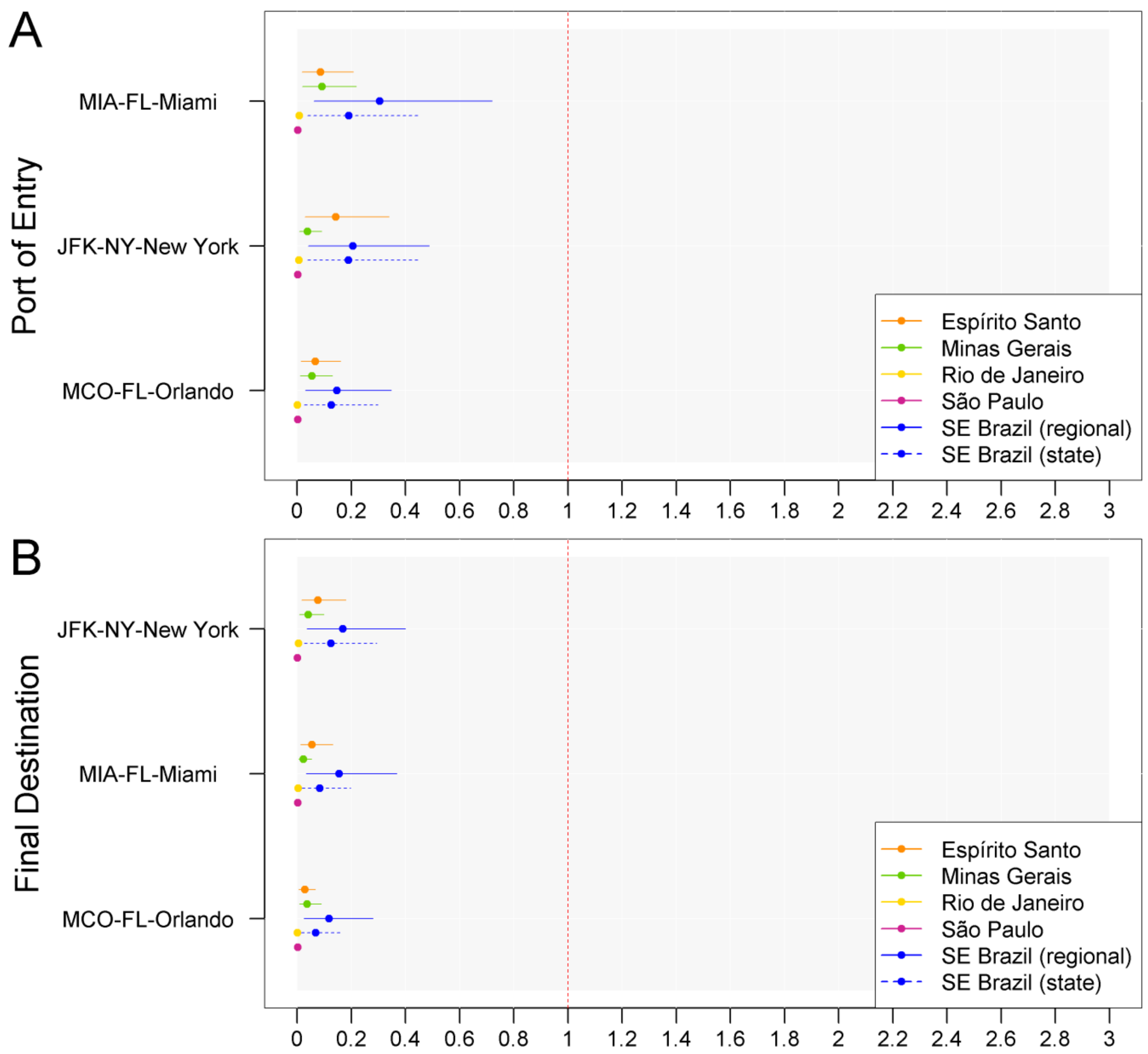

Figure S2. **Baseline risk of YFV importation from US and international travelers assuming 70% vaccination coverage of US and international travelers, 2016–2017 outbreak.** Mean and 95% confidence interval of the estimated number of YFV-infected US and international (non-Brazilian and non-US) travelers ($R_{S,A}^{W_S}$) entering the United States at the specified ports of entry (A) and final destination airports (B) during the 2016–2017 YF outbreak. These estimates were obtained assuming 70% vaccination coverage of US and international travelers to Brazil.

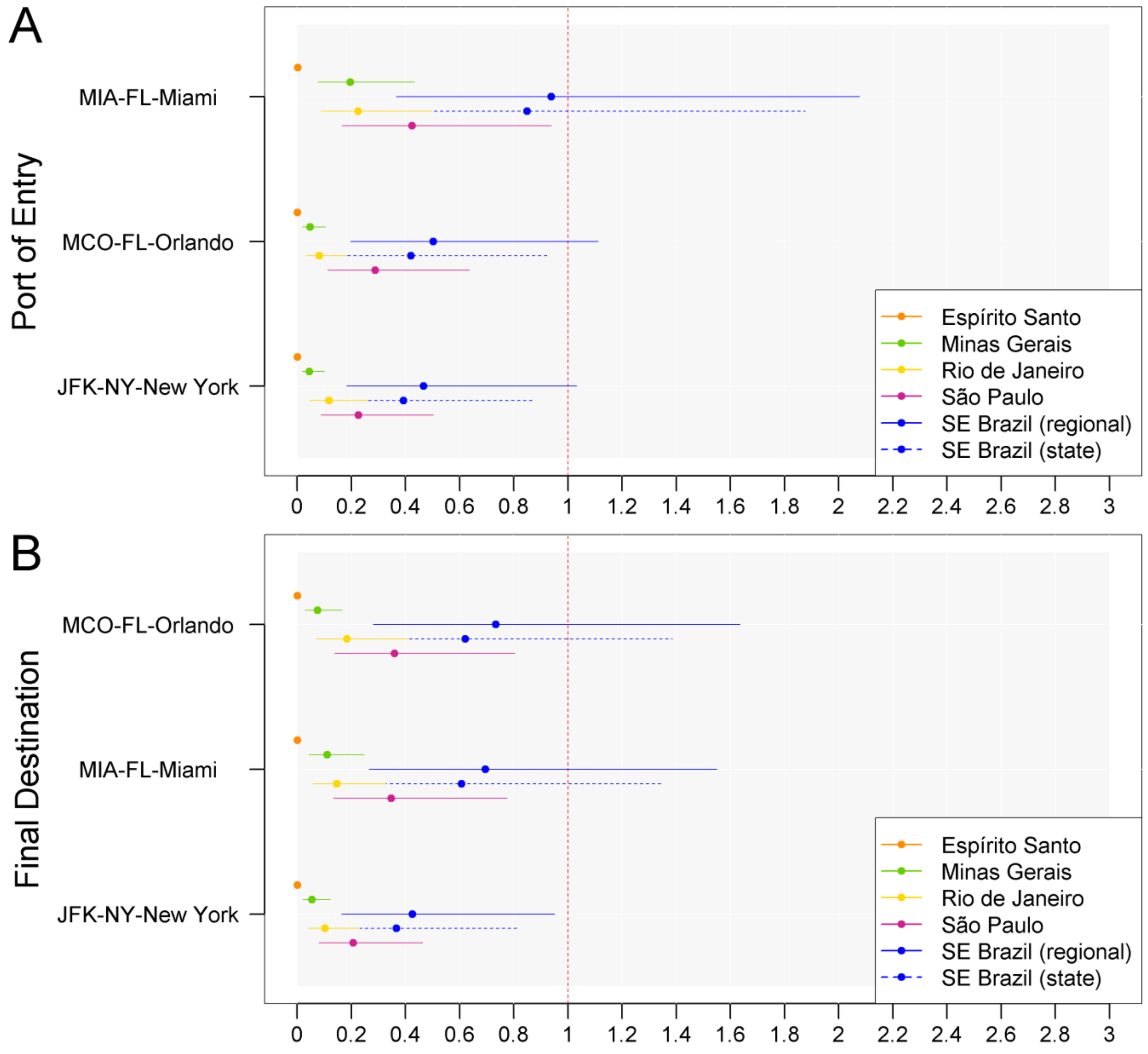

Figure S3. Baseline risk of YFV importation from Brazilian travelers, 2017–2018 outbreak. Mean and 95% confidence interval of the estimated number of YFV-infected Brazilian travelers ($E_{S,A}^{W_S}$) entering the United States at the specified ports of entry (A) and final destination airports (B) during the 2017–2018 YF outbreak. Only US airports with an upper 95% confidence limit of the total number of YFV importations exceeding 0.5 over all states (Southeast Brazil) are shown.

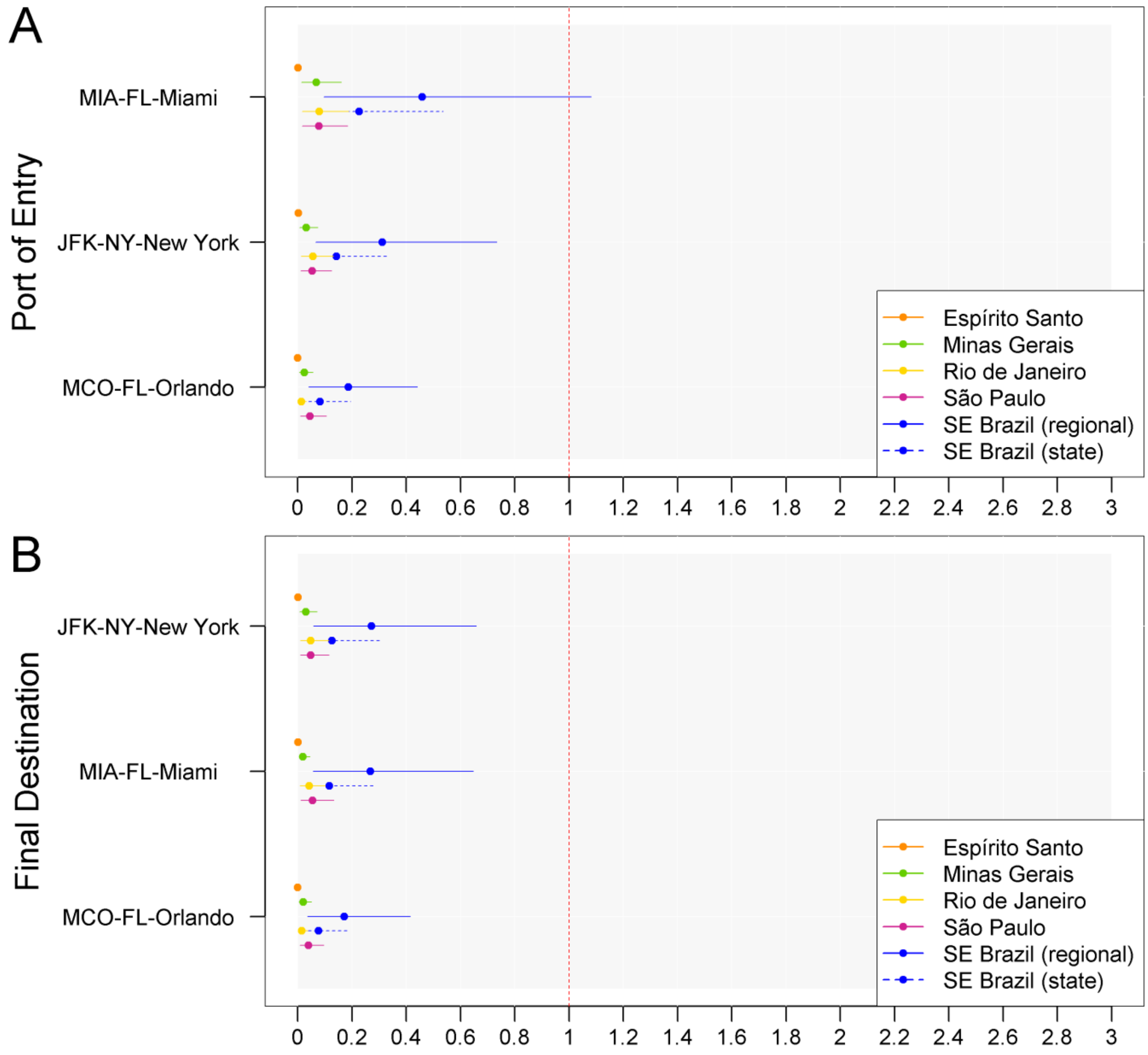

Figure S4. Baseline risk of YFV importation from US and international travelers assuming 70% vaccination coverage of US and international travelers, 2017–2018 outbreak. Mean and 95% confidence interval of the estimated number of YFV-infected US and international (non-Brazilian and non-US) travelers ($R_{S,A}^{W_S}$) entering the United States at the specified ports of entry (A) and final destination airports (B) during the 2017–2018 YF outbreak. These estimates were obtained assuming 70% vaccination coverage of US and international travelers to Brazil.

3. Sensitivity analysis of yellow fever virus vaccination coverage among US travelers

3.1 Testing the effect of extreme vaccination coverages of US and international travelers to Brazil

In the main analysis, we assumed that 70% of the US and international travelers to Brazil receive YFV vaccination (3). In this section, we show the effect of assuming extreme vaccination coverages (i.e., 0 and 100%) on the estimated number of US travelers and YFV importations (comprising both Brazilian travelers and US and international travelers) separately, for the 2016–2017 (Figures S5–S8) and 2017–2018 (Figures S9–S12) outbreaks.

3.1.1 Estimates for the 2016–2017 YF outbreak

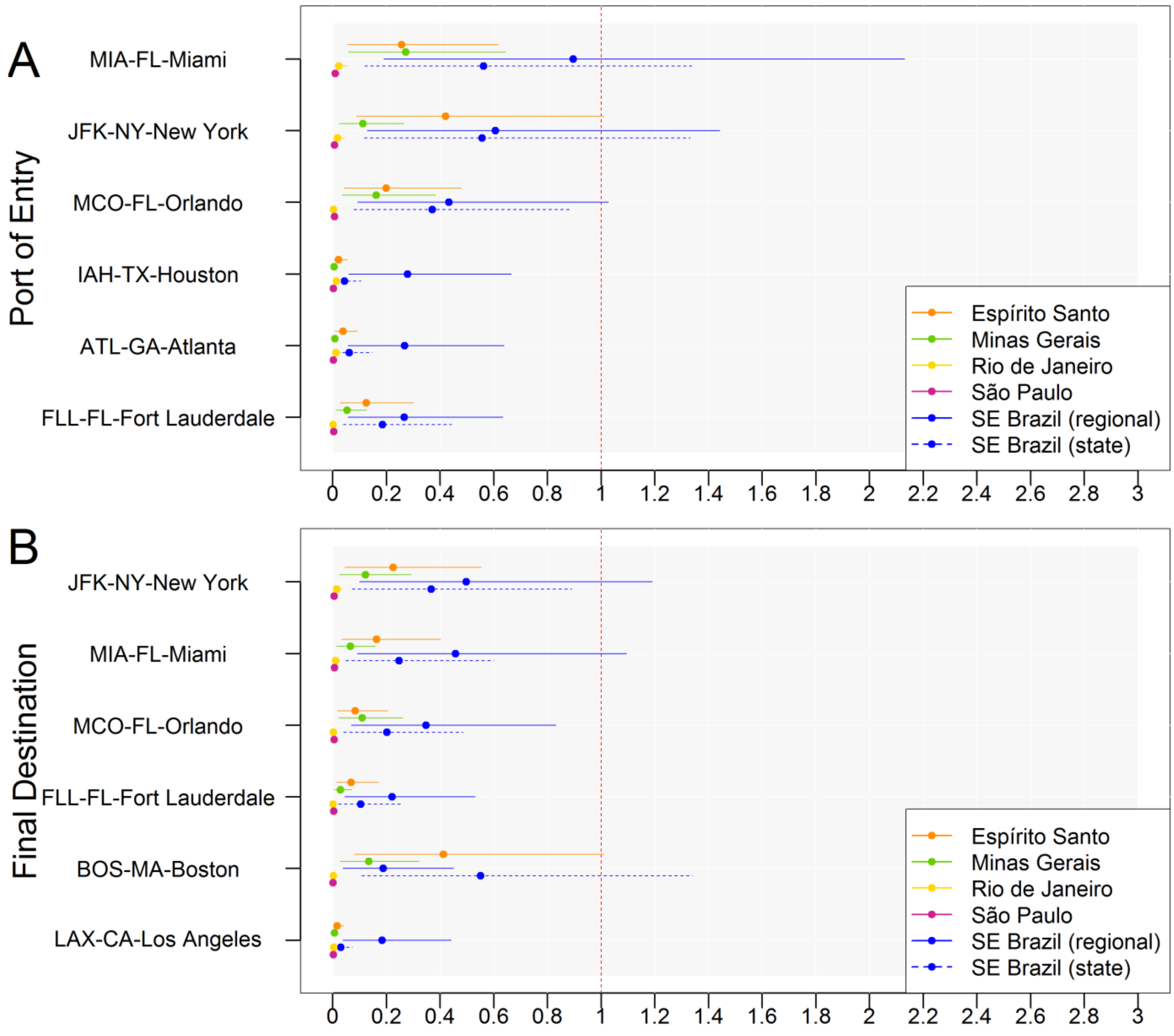

Figure S5. Sensitivity analysis: risk of YFV importation from US and international travelers assuming no (0%) vaccination coverage of US and international travelers, 2016–2017 outbreak. Mean and 95% confidence interval of the estimated number of YFV-infected US and international (non-Brazilian and non-US) travelers ($R_{S,A}^{W_S}$) entering the United States at the specified ports of entry (A) and final destination airports (B) during the 2016–2017 YF outbreak. These estimates were obtained assuming no (0%) vaccination coverage of US and international travelers to Brazil.

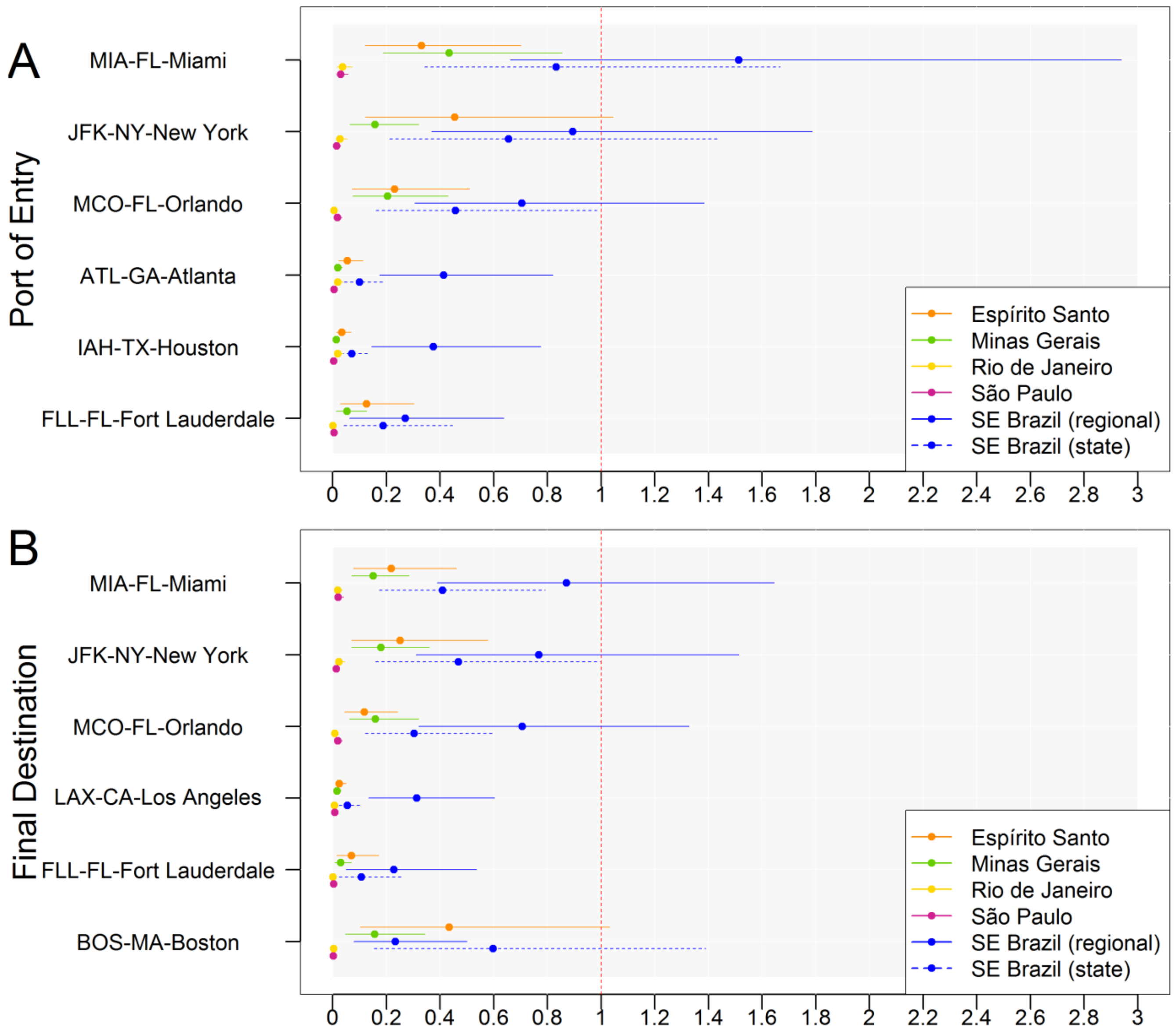

Figure S6. Sensitivity analysis: risk of YFV importation assuming no (0%) vaccination coverage of US and international travelers, 2016–2017 outbreak. Mean and 95% confidence interval of the estimated total number of YFV importations ($I_{S,A}^{W_S}$, comprising Brazilian, US and international travelers) entering the United States at the specified ports of entry (A) and final destination airports (B) during the 2016–2017 YF outbreak. These estimates were obtained assuming no (0%) vaccination coverage of US and international travelers to Brazil.

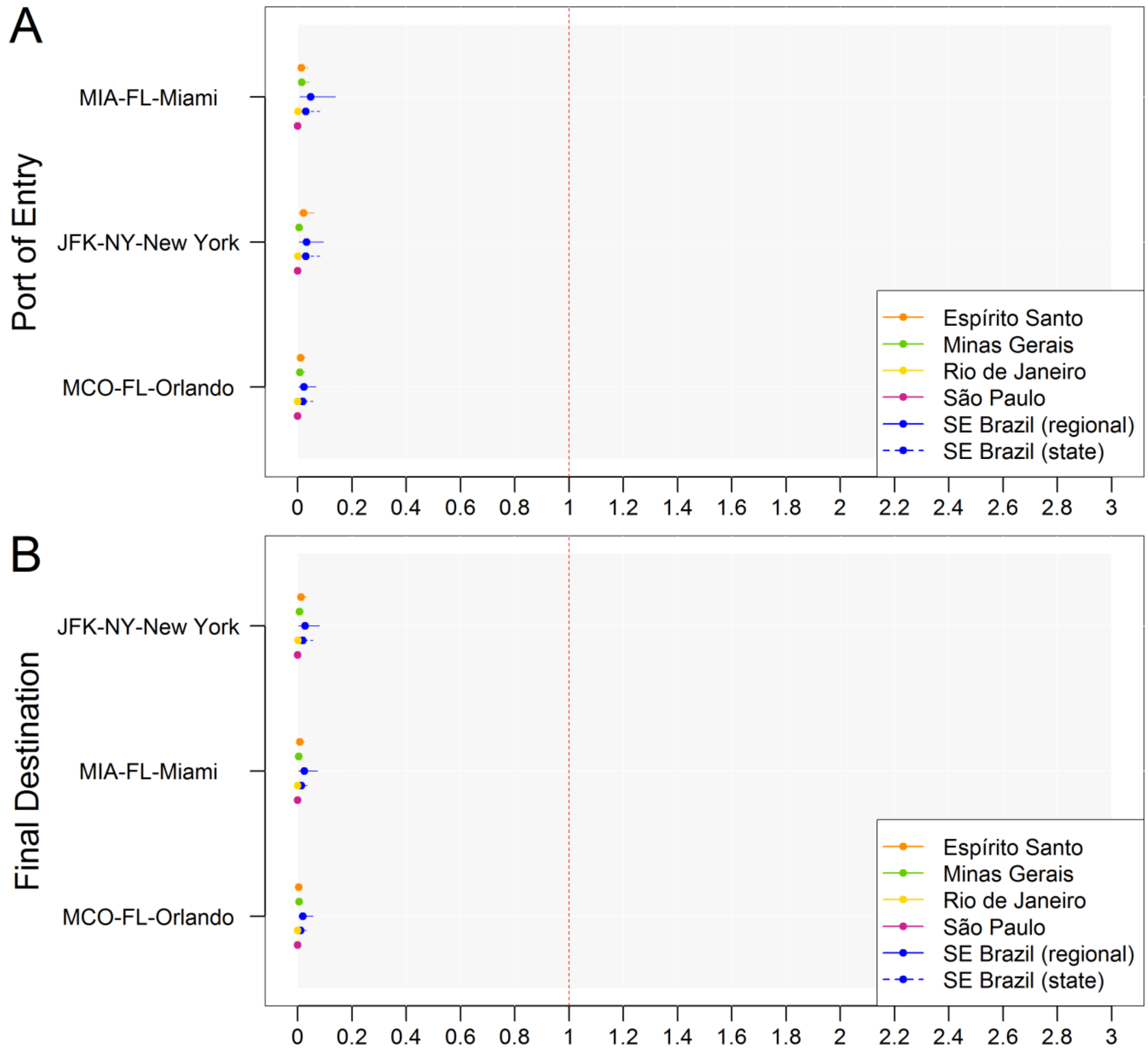

Figure S7. Sensitivity analysis: risk of YFV importation from US and international travelers assuming complete (100%) vaccination coverage of US and international travelers, 2016–2017 outbreak. Mean and 95% confidence interval of the estimated number of YFV-infected US and international (non-Brazilian and non-US) travelers ($R_{S,A}^{W_S}$) entering the United States at the specified ports of entry (A) and final destination airports (B) during the 2016–2017 YF outbreak. These estimates were obtained assuming complete (100%) vaccination coverage of US and international travelers to Brazil.

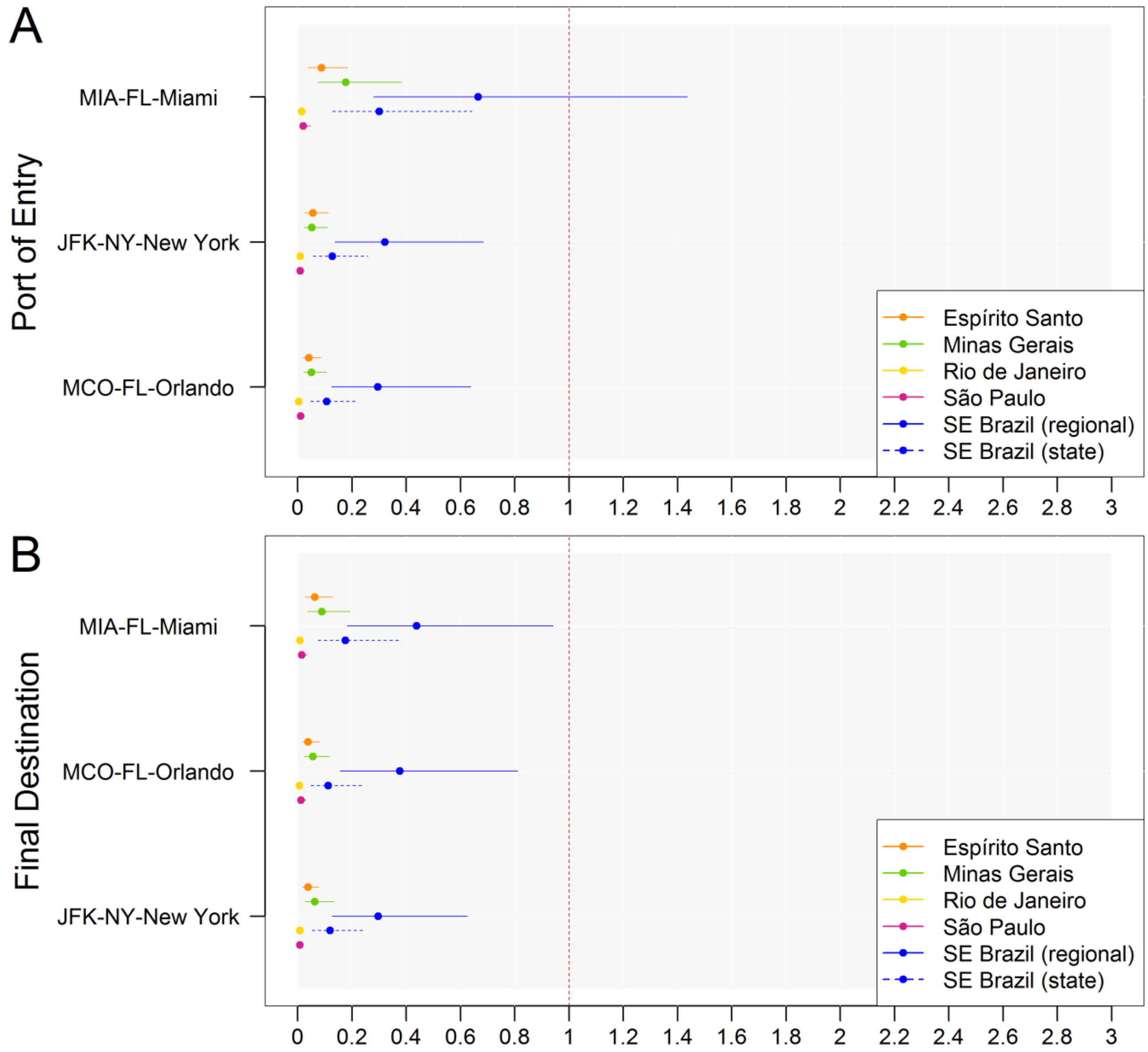

Figure S8. Sensitivity analysis: risk of YFV importation assuming complete (100%) vaccination coverage of US and international travelers, 2016–2017 outbreak. Mean and 95% confidence interval of the estimated total number of YFV importations ($I_{S,A}^{W_S}$, comprising Brazilian, US and international travelers) entering the United States at the specified ports of entry (A) and final destination airports (B) during the 2016–2017 YF outbreak. These estimates were obtained assuming complete (100%) vaccination coverage of US and international travelers to Brazil.

3.1.2 Estimates for the 2017–2018 YF outbreak

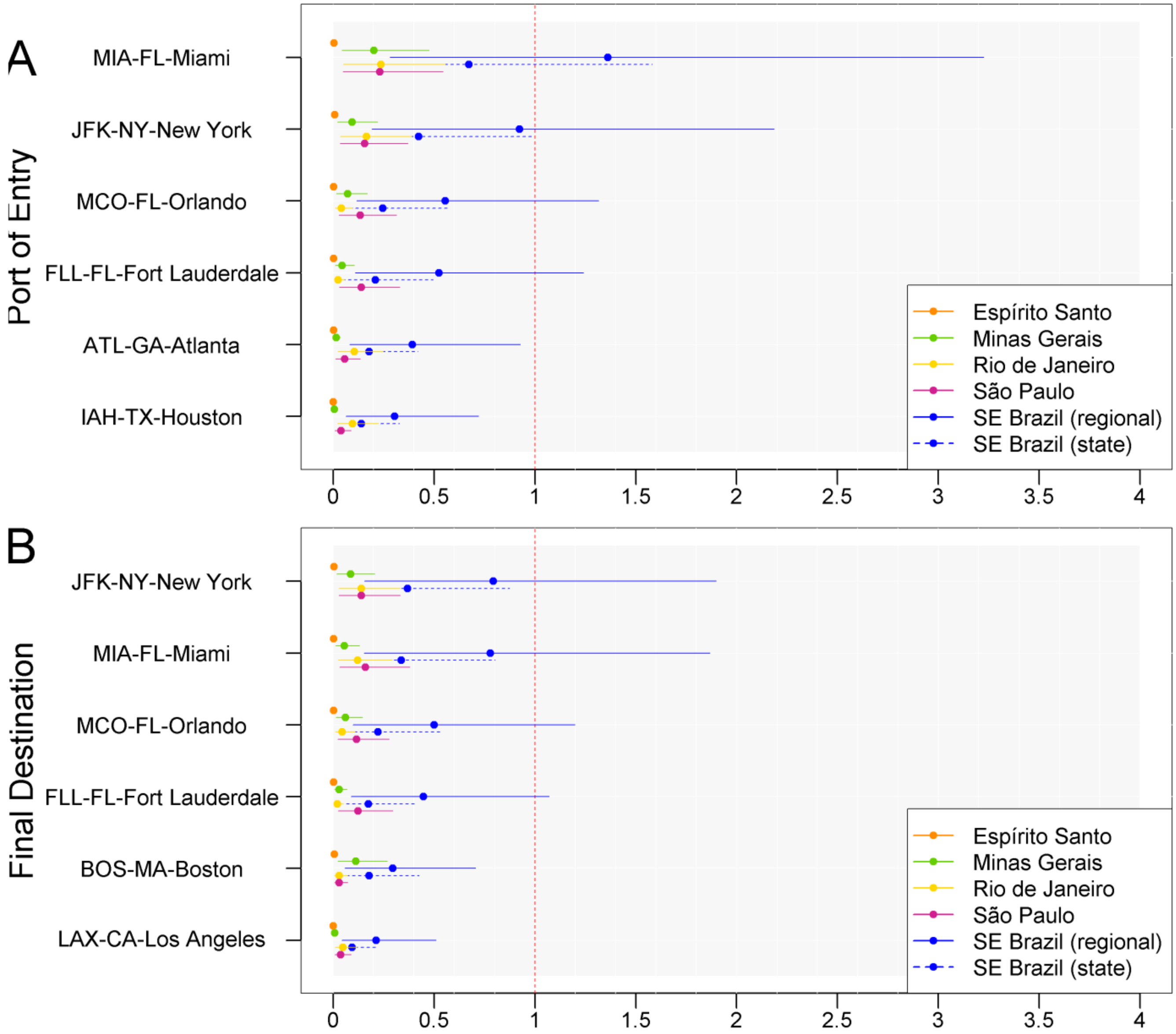

Figure S9. Sensitivity analysis: risk of YFV importation from US and international travelers assuming no (0%) vaccination coverage of US and international travelers, 2017–2018 outbreak. Mean and 95% confidence interval of the estimated number of YFV-infected US and international (non-Brazilian and non-US) travelers ($R_{S,A}^{W_S}$) entering the United States at the specified ports of entry (A) and final destination airports (B) during the 2017–2018 YF outbreak. These estimates were obtained assuming no (0%) vaccination coverage of US and international travelers to Brazil.

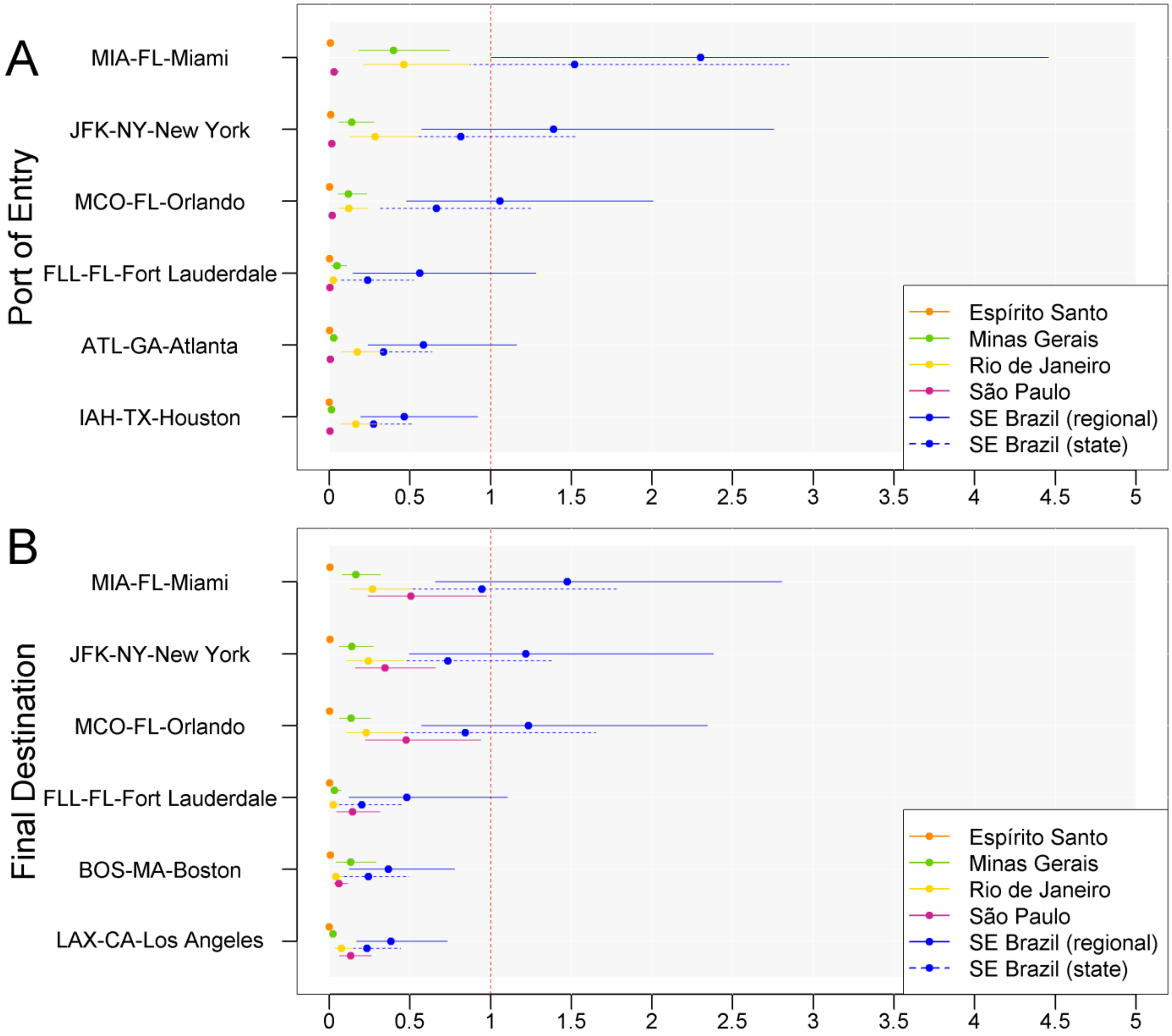

Figure S10. Sensitivity analysis: risk of YFV importation assuming no (0%) vaccination coverage of US and international travelers, 2017–2018 outbreak. Mean and 95% confidence interval of the estimated total number of YFV importations ($I_{S,A}^{W_S}$, comprising Brazilian, US and international travelers) entering the United States at the specified ports of entry (A) and final destination airports (B) during the 2017–2018 YF outbreak. These estimates were obtained assuming no (0%) vaccination coverage of US and international travelers to Brazil.

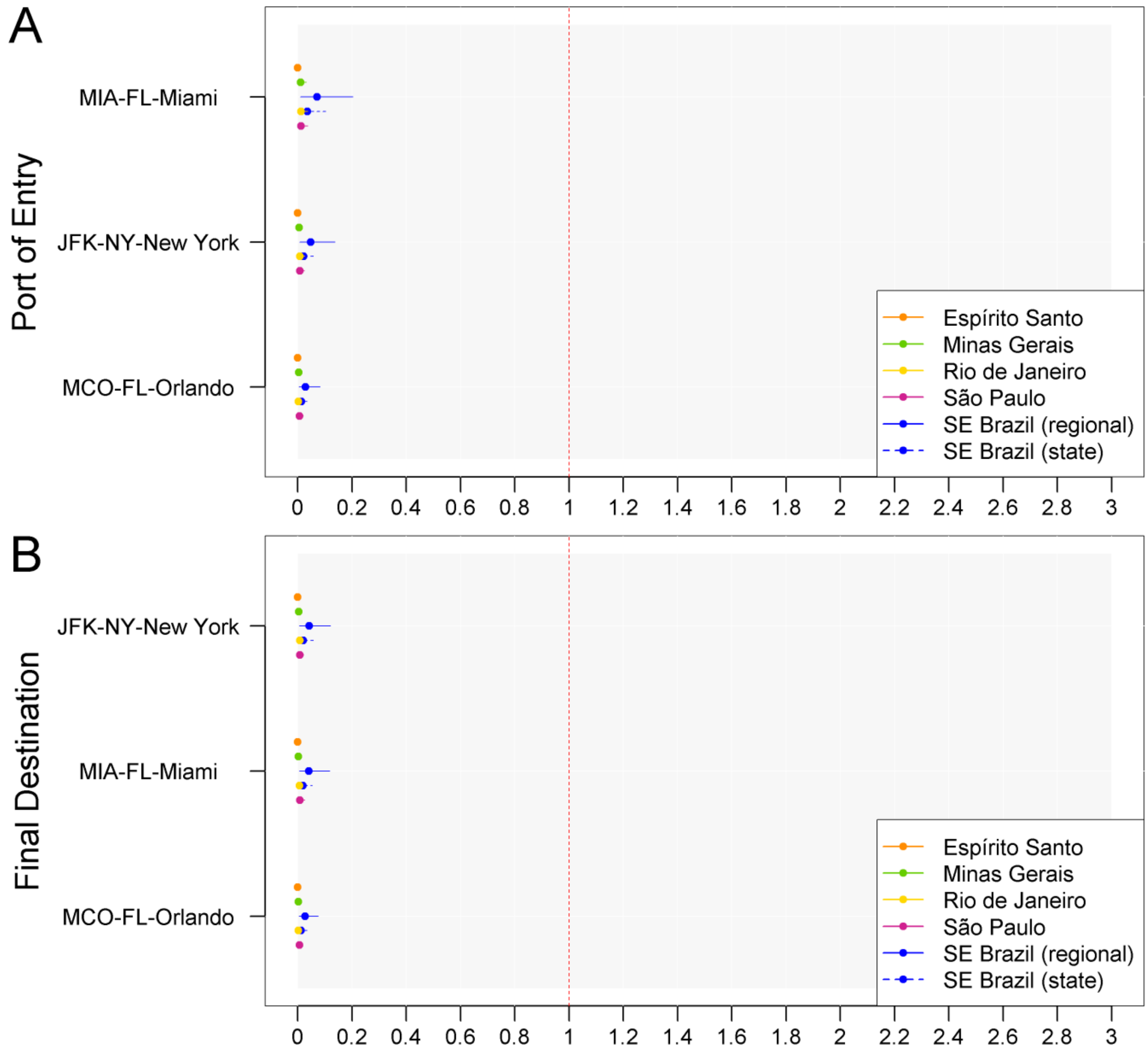

Figure S11. Sensitivity analysis: risk of YFV importation from US and international travelers assuming complete (100%) vaccination coverage of US and international travelers, 2017–2018 outbreak. Mean and 95% confidence interval of the estimated number of YFV-infected US and international (non-Brazilian and non-US) travelers ($R_{S,\mathrm{A}}^{W_S}$) entering the United States at the specified ports of entry (A) and final destination airports (B) during the 2017–2018 YF outbreak. These estimates were obtained assuming complete (100%) vaccination coverage of US and international travelers to Brazil.

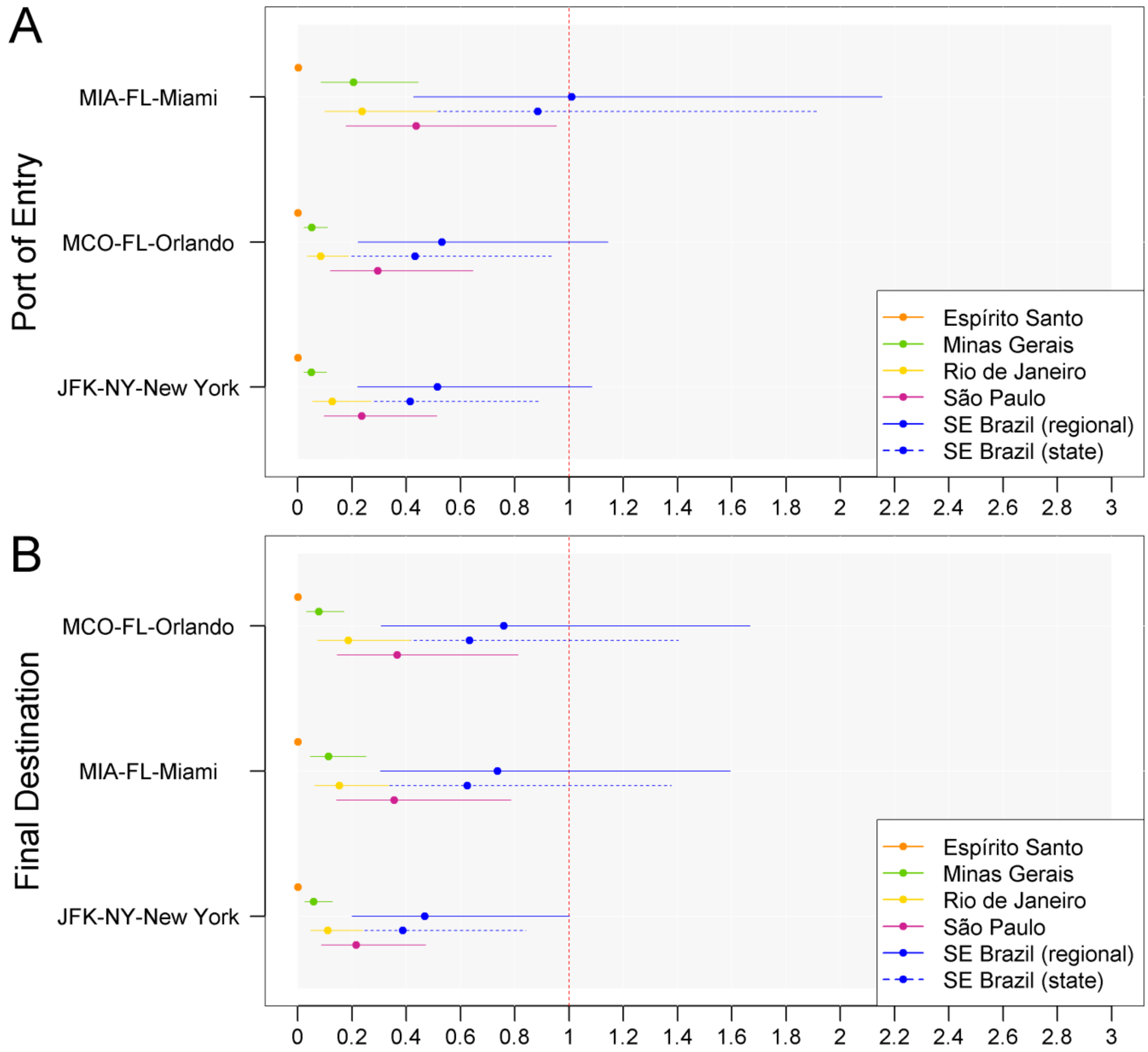

Figure S12. Sensitivity analysis: risk of YFV importation assuming complete (100%) vaccination coverage of US and international travelers, 2017–2018 outbreak. Mean and 95% confidence interval of the estimated total number of YFV importations ($I_{S,A}^{W_S}$, comprising Brazilian, US and international travelers) entering the United States at the specified ports of entry (A) and final destination airports (B) during the 2017–2018 YF outbreak. These estimates were obtained assuming complete (100%) vaccination coverage of US and international travelers to Brazil.

4. Counterfactual analysis on yellow fever virus vaccination coverage among residents of Brazil

We analyzed the effect of higher and lower YFV vaccination coverages in Southeast Brazil on the risk of YFV importation into the United States by exploring hypothetical scenarios.

4.1 2016–2017 YF outbreak

Vaccination campaigns were conducted in Espírito Santo, Minas Gerais, São Paulo, and Rio de Janeiro from the start of the 2016–2017 YF outbreak to limit the spread of the disease (5). In this section, we analyzed the effects that higher vaccination coverage during the 2016–2017 YFV immunization campaign as well as no vaccination would have had on the risk of YFV importation into the United States.

In Scenario 1 we assumed a 10% increase in the number of subjects with YFV vaccine-induced immunity in all states from the corresponding estimate obtained in 2016–2017 YF ($pop_{S,V}$). In Scenarios 2 and 3 we assumed a 20% increase in the number of subjects with YF vaccine-induced immunity from the corresponding estimate obtained in 2016–2017 ($pop_{S,V}$) in all Southeastern states of Brazil except Minas Gerais, where we assumed a 30% and 80% increase, respectively. The larger increases assumed in Scenarios 2 and 3 for the state of Minas Gerais reflect the larger number of reported YFV cases in the state, and hence the larger impact that increases in YFV vaccine-induced protection would have on the risk of YFV spread. In Scenario 4, we explored the effect that absence of YFV vaccination in Southeast Brazil in 2016–2017 would have had on the risk of YFV importation in the United States that year.

In each scenario, we used the counterfactual number of subjects immunized against YFV ($\widetilde{pop}_{S,V}$) to calculate the counterfactual cumulative number of confirmed YF cases ($\tilde{C}_{S,W_s}$), having assumed proportionality between the changes in the cumulative number of confirmed YFV cases and number of subjects susceptible to YFV:

$$\tilde{C}_{S,W_s} = \hat{C}_{S,W_s} \frac{(pop_S - \widetilde{pop}_{S,V})}{(pop_S - pop_{S,V})}$$

We then used the counterfactual number of confirmed YF cases ($\tilde{C}_{S,W_s}$) to estimate the risk of importation due to Brazilian travelers and US and international travelers (note that because of the assumed proportionality between changes in the cumulative number of confirmed YFV cases and vaccination rates, the estimated number of returning US and international travelers in the counterfactual analysis is equal to the estimated number of returning US and international travelers obtained in the main analysis). The counterfactual number of subjects immunized against YFV for Southeast Brazil was given by the sum of the counterfactual number of subjects immunized against YFV in each state in Southeast Brazil. This analysis was conducted under the baseline (70%) vaccination coverage for US and international travelers visiting Brazil. The four scenarios analyzed in this section are summarized in Table S1.

Figures S15–S18 show the estimated counterfactual total number of YFV importations (comprising Brazilian, US and international travelers) in the specified US ports of entry and final destination airports during the 2016–2017 YF outbreak, obtained under Scenarios 1–4, respectively.

Table S1. Vaccination coverage scenarios explored in the counterfactual analysis.

State	Scenario 1 $pop_{S,\tilde{V}} =$	Scenario 2 $pop_{S,\tilde{V}} =$	Scenario 3 $pop_{S,\tilde{V}} =$	Scenario 4 $pop_{S,\tilde{V}} =$
Espírito Santo	$= pop_{S,V} \cdot (1+10\%)$	$= pop_{S,V} \cdot (1+20\%)$	$= pop_{S,V} \cdot (1+20\%)$	0
Minas Gerais	$= pop_{S,V} \cdot (1+10\%)$	$= pop_{S,V} \cdot (1+30\%)$	$= pop_{S,V} \cdot (1+80\%)$	0
Rio de Janeiro	$= pop_{S,V} \cdot (1+10\%)$	$= pop_{S,V} \cdot (1+20\%)$	$= pop_{S,V} \cdot (1+20\%)$	0
São Paulo	$= pop_{S,V} \cdot (1+10\%)$	$= pop_{S,V} \cdot (1+20\%)$	$= pop_{S,V} \cdot (1+20\%)$	0

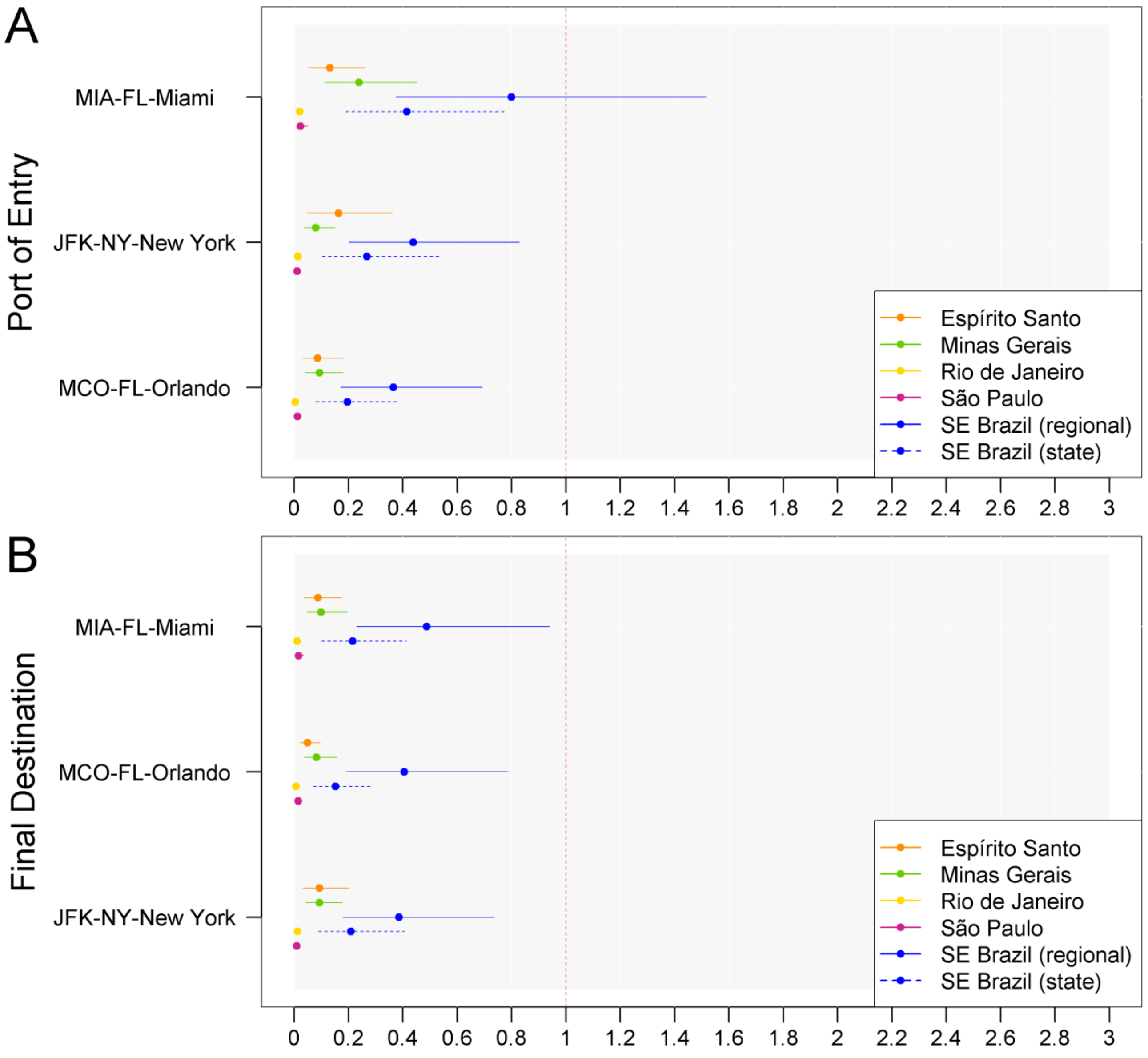

Figure 5S13. Counterfactual analysis: risk of YFV importation assuming counterfactual scenario 1, 2016–2017 outbreak. Mean and 95% confidence interval of the estimated counterfactual total number of YFV importations ($I_{S,A}^{W_S}$, comprising Brazilian, US and international travelers) entering the United States at the specified ports of entry (A) and final destination airports (B) during the 2016–2017 YF outbreak, having assumed a 10% increase in the vaccination coverage in Espírito Santo, Minas Gerais, São Paulo, and Rio de Janeiro (Scenario 1 of Table S4). These estimates were obtained assuming 70% vaccination coverage of US and international travelers to Brazil.

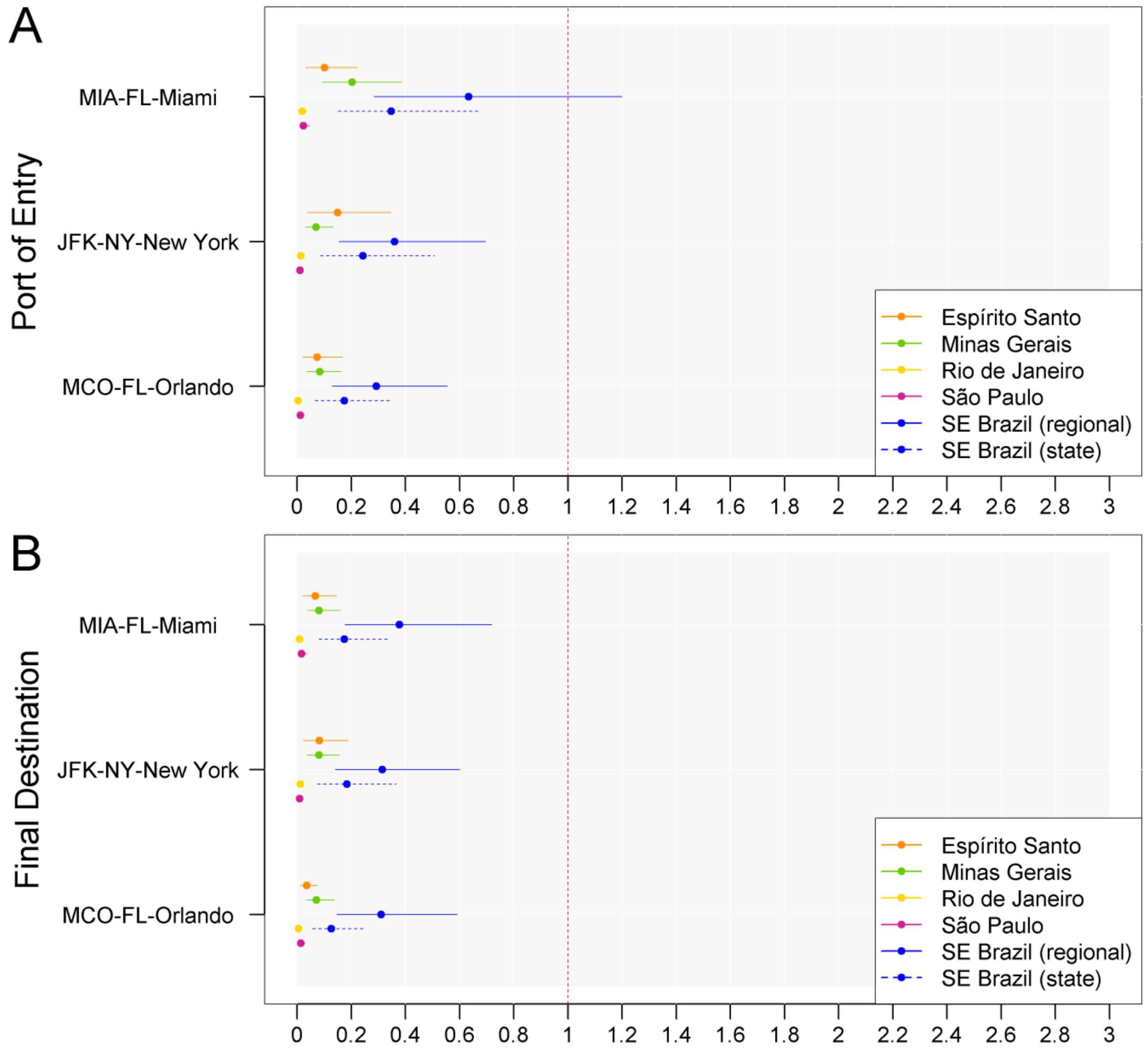

Figure S14. Counterfactual analysis: risk of YFV importation assuming counterfactual scenario 2, 2016–2017 outbreak. Mean and 95% confidence interval of the estimated counterfactual total number of YFV importations ($I_{S,A}^{W_S}$, comprising Brazilian, US and international travelers) entering the United States at the specified ports of entry (A) and final destination airports (B) during the 2016–2017 YF outbreak, having assumed a 20% increase in the vaccination coverage in Espírito Santo, São Paulo and Rio de Janeiro and a 30% increase in the vaccination coverage in Minas Gerais, (Scenario 2 of Table S4). These estimates were obtained assuming 70% vaccination coverage of US and international travelers to Brazil.

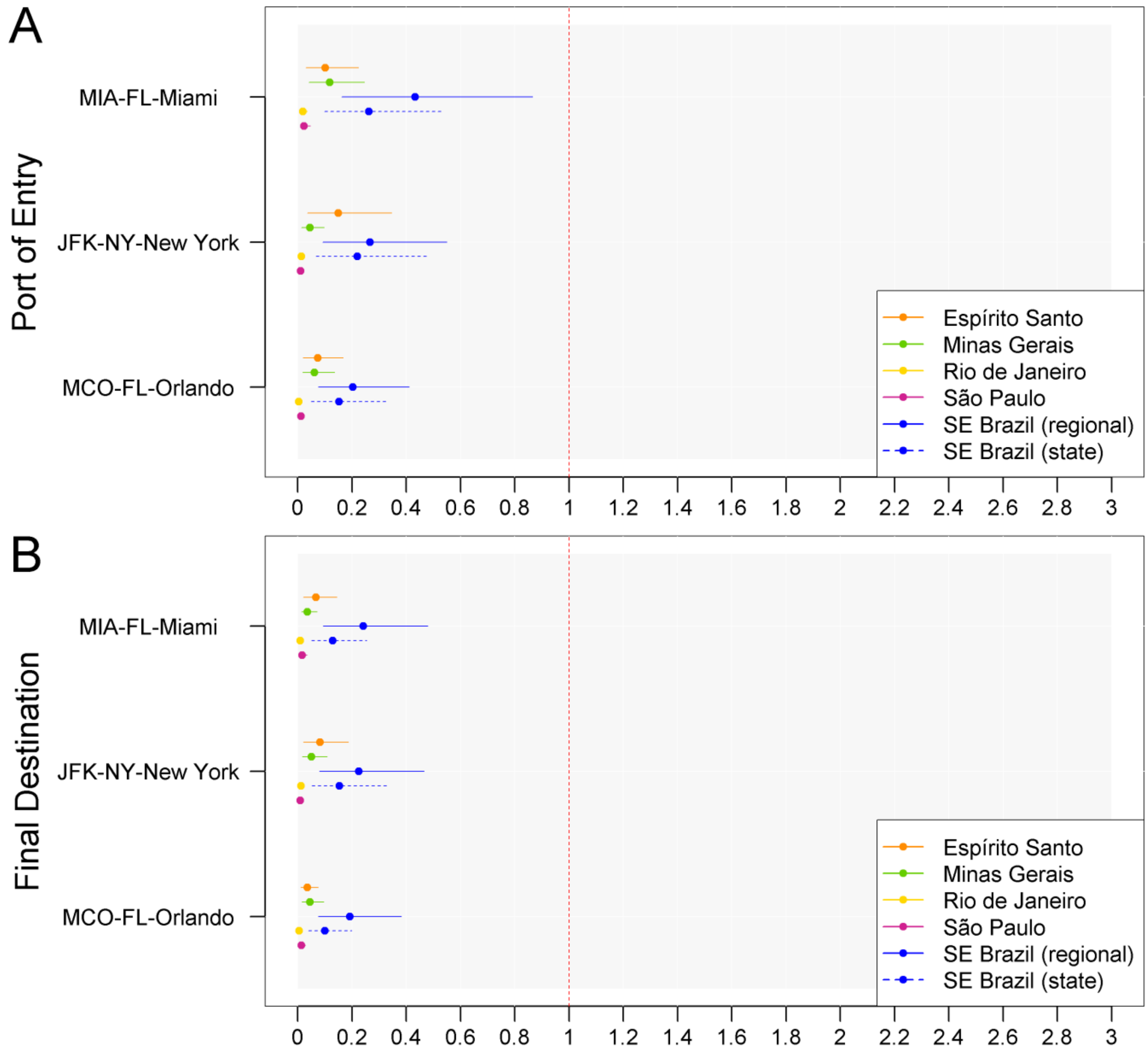

Figure S15. Counterfactual analysis: risk of YFV importation assuming counterfactual scenario 3, 2016–2017 outbreak. Mean and 95% confidence interval of the estimated counterfactual total number of YFV importations ($I_{S,A}^{W_S}$, comprising Brazilian, US and international travelers) entering the United States at the specified ports of entry (A) and final destination airports (B) during the 2016–2017 YF outbreak having assumed a 20% increase in the vaccination coverage in Espírito Santo, São Paulo, and Rio de Janeiro and an 80% increase in the vaccination coverage in Minas Gerais (Scenario 3 of Table S4). These estimates were obtained assuming 70% vaccination coverage of US and international travelers to Brazil.

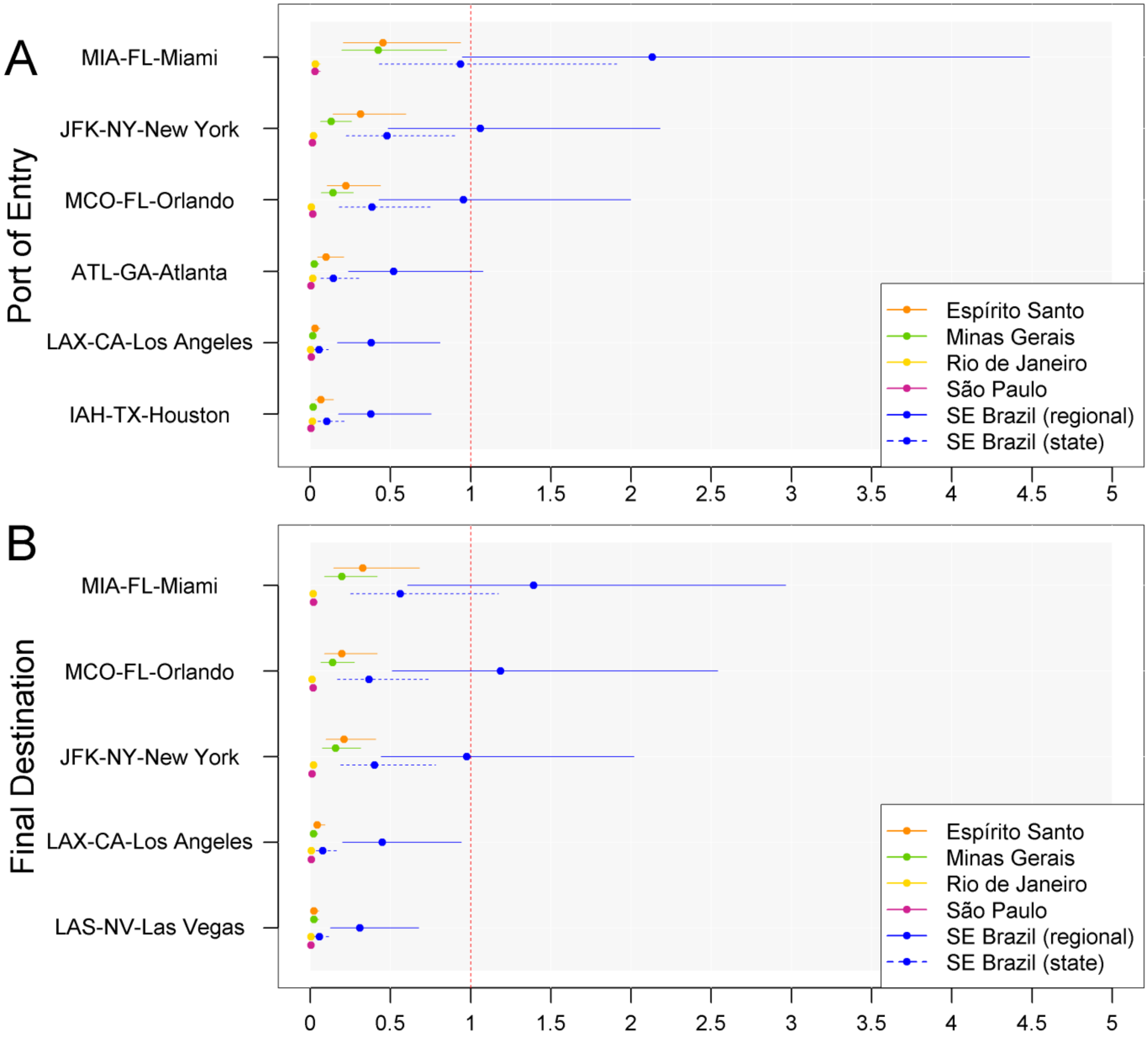

Figure S16.Counterfactual analysis: risk of YFV importation assuming counterfactual scenario 4, 2016–2017 outbreak. Mean and 95% confidence interval of the estimated counterfactual total number of YFV importations ($I_{S,A}^{W_S}$, comprising Brazilian travelers, US and international travelers) entering the United States at the specified ports of entry (A) and final destination airports (B) during the 2016–2017 YF outbreak, having assumed no vaccination in Southeast Brazil (Scenario 4 of Table S4). These estimates were obtained assuming 70% vaccination coverage of US and international travelers to Brazil.

4.2 2017–2018 YF outbreak

On January 25, 2018, in response to the 2017–2018 YF outbreak, the states of São Paulo and Rio de Janeiro started a new, extensive vaccination campaign targeting all residents older than 9 months with no proof of previous YFV vaccination (4).

In this section, we explore the effect that the 2018 vaccination campaign had on the risk of YFV importation in the United States by exploring the counterfactual number of YFV importations that would have been observed if the 2018 vaccination campaign was not conducted (i.e., by setting $pop_{S,V2018} = 0$).

Figure S19 shows the counterfactual total number of YFV importations in the United States that we estimated having assumed no vaccination in 2018.

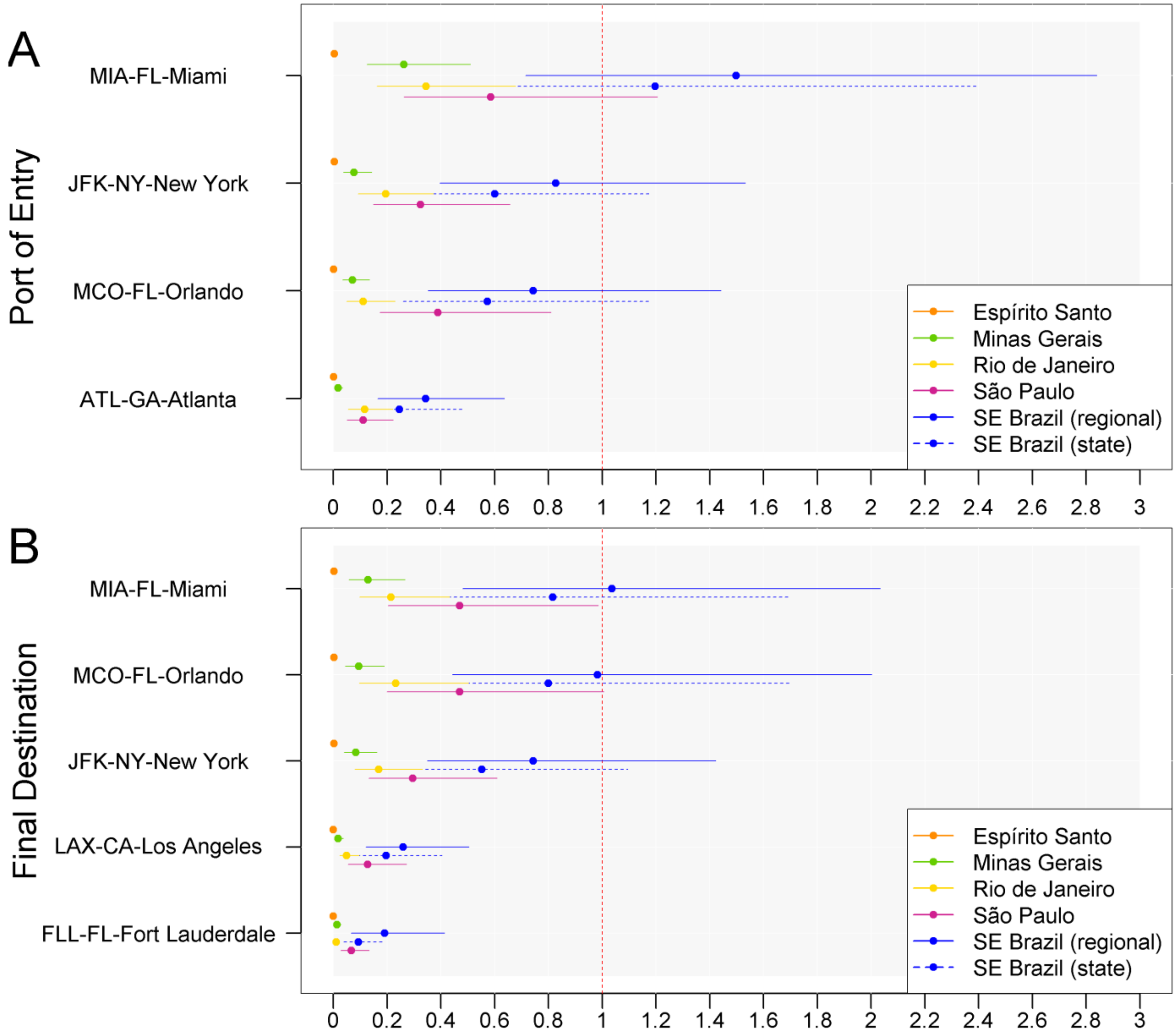

Figure S17. **Counterfactual analysis: risk of YFV importation assuming no vaccination in Brazil in 2018, 2016–2017 outbreak.** Mean and 95% confidence interval of the estimated counterfactual total number of YFV importations ($I_{S,A}^{W_S}$, comprising Brazilian, US and international travelers) entering the United States at the specified ports of entry (A) and final destination airports (B) during the 2017–2018 YF outbreak, having assumed no vaccination in 2018. These estimates were obtained assuming 70% vaccination coverage of US and international travelers to Brazil.

5. Summary tables

5.1 2016–2017 YF outbreak

	Baseline		Sensitivity Analysis			
	70% vaccination coverage travelers		No vaccination coverage of travelers		100% vaccination coverage travelers	
	SE Brazil (regional)	SE Brazil (state)	SE Brazil (regional)	SE Brazil (state)	SE Brazil (regional)	SE Brazil (state)
**MIA-FL-Miami**	0.92 (0.43, 1.77)	0.46 (0.21, 0.88)	1.51 (0.66, 2.94)	0.83 (0.34, 1.66)	0.66 (0.28, 1.44)	0.30 (0.13, 0.64)
**JFK-NY-New York**	0.49 (0.23, 0.94)	0.29 (0.12, 0.56)	0.89 (0.37, 1.79)	0.65 (0.21, 1.44)	0.32 (0.13, 0.68)	0.13 (0.06, 0.26)
**MCO-FL-Orlando**	0.42 (0.20, 0.80)	0.21 (0.09, 0.41)	0.70 (0.30, 1.38)	0.46 (0.16, 0.98)	0.29 (0.12, 0.64)	0.10 (0.05, 0.22)
**ATL-GA-Atlanta**	0.24 (0.11, 0.45)	0.06 (0.03, 0.11)	0.41 (0.17, 0.82)	0.10 (0.04, 0.20)	0.16 (0.07, 0.34)	0.04 (0.02, 0.09)
**IAH-TX-Houston**	0.19 (0.09, 0.36)	0.04 (0.02, 0.08)	0.38 (0.14, 0.78)	0.07 (0.03, 0.14)	0.11 (0.05, 0.23)	0.03 (0.01, 0.06)
**FLL-FL-Fort Lauderdale**	0.10 (0.02, 0.22)	0.07 (0.01, 0.15)	0.27 (0.06, 0.64)	0.19 (0.04, 0.45)	0.02 (0.01, 0.05)	0.01 (0.00, 0.03)
**LAX-CA-Los Angeles**	0.16 (0.07, 0.31)	0.03 (0.01, 0.05)	0.25 (0.11, 0.48)	0.05 (0.02, 0.09)	0.12 (0.05, 0.26)	0.02 (0.01, 0.04)

Table S2. **Risk of YFV importation into US first ports of entry from Southeast Brazil: baseline and sensitivity analyses, 2016-2017 outbreak.** Mean and 95% confidence interval of the estimated total number of YFV importations ($I_{S,A}^{W_S}$, comprising both Brazilian travelers and US and international travelers) entering the United States at the specified ports of entry during the 2016–2017 YF outbreak. Only estimates for Southeast Brazil at the regional and state level are included. SE Brazil = Southeast Brazil.

	Counterfactual Analysis							
	Scenario 1 (10% increase in vaccination coverage in ES, MG, RJ, SP)		Scenario 2 (20% increase in vaccination coverage in ES, MG, RJ, SP)		Scenario 3 (20% increase in vaccination coverage in ES, RJ, SP and 30% increase in vaccination coverage in MG)		Scenario 4 (No vaccination coverage in ES, MG, RJ, SP)	
	SE Brazil (regional)	SE Brazil (state)	SE Brazil (regional)	SE Brazil (state)	SE Brazil (regional)	SE Brazil (state)	SE Brazil (regional)	SE Brazil (state)
**MIA-FL-Miami**	0.80 (0.37, 1.52)	0.41 (0.19, 0.78)	0.63 (0.28, 1.20)	0.35 (0.15, 0.67)	0.43 (0.16, 0.87)	0.26 (0.10, 0.53)	2.13 (0.94, 4.48)	0.94 (0.43, 1.90)
**JFK-NY-New York**	0.44 (0.20, 0.83)	0.27 (0.10, 0.54)	0.36 (0.15, 0.69)	0.24 (0.08, 0.51)	0.27 (0.09, 0.55)	0.22 (0.07, 0.48)	1.06 (0.48, 2.18)	0.48 (0.22, 0.91)
**MCO-FL-Orlando**	0.37 (0.17, 0.69)	0.19 (0.08, 0.38)	0.29 (0.13, 0.55)	0.17 (0.07, 0.35)	0.20 (0.08, 0.41)	0.15 (0.05, 0.32)	0.96 (0.43, 2.00)	0.38 (0.18, 0.75)
**ATL-GA-Atlanta**	0.21 (0.09, 0.39)	0.05 (0.02, 0.09)	0.17 (0.07, 0.32)	0.04 (0.02, 0.08)	0.12 (0.04, 0.25)	0.03 (0.01, 0.07)	0.52 (0.23, 1.08)	0.14 (0.06, 0.30)
**IAH-TX-Houston**	0.17 (0.07, 0.33)	0.04 (0.02, 0.07)	0.14 (0.06, 0.29)	0.03 (0.01, 0.06)	0.11 (0.04, 0.24)	0.02 (0.01, 0.05)	0.38 (0.17, 0.75)	0.10 (0.05, 0.22)
**FLL-FL-Fort Lauderdale**	0.09 (0.02, 0.22)	0.06 (0.01, 0.15)	0.09 (0.02, 0.22)	0.06 (0.01, 0.15)	0.09 (0.02, 0.21)	0.06 (0.01, 0.15)	0.10 (0.03, 0.23)	0.07 (0.02, 0.16)
**LAX-CA-Los Angeles**	0.14 (0.06, 0.26)	0.02 (0.01, 0.05)	0.11 (0.05, 0.20)	0.02 (0.01, 0.04)	0.07 (0.03, 0.14)	0.02 (0.01, 0.03)	0.38 (0.17, 0.81)	0.05 (0.02, 0.11)

Table S3. **Risk of YFV importation into US first ports of entry from Southeast Brazil: counterfactual analysis, 2016-2017 outbreak.** Mean and 95% confidence interval of the estimated total number of YFV importations ($I_{S,A}^{W_S}$, comprising both Brazilian travelers and US and international travelers) entering the United States at the specified ports of entry during the 2016–2017 YF outbreak. Only estimates for Southeast Brazil at the regional and state level are included. ES = Espírito Santo, MG = Minas Gerais, RJ = Rio de Janeiro, SP = São Paulo, SE Brazil = Southeast Brazil.
.

	Baseline		Sensitivity Analysis			
	70% vaccination coverage travelers		No vaccination coverage of travelers		100% vaccination coverage travelers	
	SE Brazil (regional)	SE Brazil (state)	SE Brazil (regional)	SE Brazil (state)	SE Brazil (regional)	SE Brazil (state)
**MIA-FL-Miami**	0.57 (0.27, 1.10)	0.25 (0.12, 0.47)	0.88 (0.39, 1.65)	0.41 (0.17, 0.79)	0.44 (0.18, 0.94)	0.17 (0.07, 0.37)
**JFK-NY-New York**	0.44 (0.21, 0.83)	0.22 (0.10, 0.43)	0.76 (0.31, 1.51)	0.47 (0.16, 1.00)	0.29 (0.13, 0.63)	0.12 (0.05, 0.25)
**MCO-FL-Orlando**	0.47 (0.22, 0.93)	0.17 (0.08, 0.32)	0.71 (0.32, 1.33)	0.30 (0.12, 0.60)	0.37 (0.15, 0.81)	0.11 (0.05, 0.24)
**LAX-CA-Los Angeles**	0.19 (0.09, 0.37)	0.03 (0.02, 0.07)	0.31 (0.11, 0.60)	0.05 (0.02, 0.10)	0.14 (0.06, 0.30)	0.03 (0.01, 0.06)
**FLL-FL-Fort Lauderdale**	0.08 (0.02, 0.18)	0.04 (0.01, 0.09)	0.23 (0.05, 0.54)	0.11 (0.02, 0.25)	0.02 (0.01, 0.04)	0.01 (0.00, 0.02)
**BOS-MA-Boston**	0.11 (0.05, 0.21)	0.23 (0.08, 0.50)	0.23 (0.08, 0.50)	0.60 (0.15, 1.39)	0.06 (0.02, 0.11)	0.07 (0.03, 0.15)
**LAS-NV-Las Vegas**	0.11 (0.05, 0.23)	0.02 (0.01, 0.05)	0.14 (0.07, 0.27)	0.03 (0.01, 0.06)	0.10 (0.04, 0.22)	0.02 (0.01, 0.05)

Table S4. **Risk of YFV importation into US final destination airports from Southeast Brazil: baseline and sensitivity analyses, 2016-2017 outbreak.** Mean and 95% confidence interval of the estimated total number of YFV importations ($I_{S,\mathrm{A}}^{W_S}$, comprising both Brazilian travelers and US and international travelers) entering the United States at the specified final destination airports during the 2016–2017 YF outbreak. Only estimates for Southeast Brazil at the regional and state level are included. SE Brazil = Southeast Brazil.

	Counterfactual Analysis							
	Scenario 1 (10% increase in vaccination coverage in ES, MG, RJ, SP)		Scenario 2 (20% increase in vaccination coverage in ES, MG, RJ, SP)		Scenario 3 (20% increase in vaccination coverage in ES, RJ, SP and 30% increase in vaccination coverage in MG)		Scenario 4 (No vaccination coverage in ES, MG, RJ, SP)	
	SE Brazil (regional)	SE Brazil (state)	SE Brazil (regional)	SE Brazil (state)	SE Brazil (regional)	SE Brazil (state)	SE Brazil (regional)	SE Brazil (state)
**MIA-FL-Miami**	0.49 (0.23, 0.94)	0.21 (0.10, 0.42)	0.38 (0.18, 0.72)	0.17 (0.08, 0.33)	0.24 (0.09, 0.48)	0.13 (0.05, 0.25)	1.39 (0.60, 2.96)	0.56 (0.25, 1.17)
**JFK-NY-New York**	0.39 (0.18, 0.74)	0.21 (0.09, 0.41)	0.31 (0.14, 0.60)	0.18 (0.07, 0.37)	0.23 (0.08, 0.47)	0.15 (0.05, 0.33)	0.98 (0.44, 2.02)	0.40 (0.19, 0.78)
**MCO-FL-Orlando**	0.41 (0.19, 0.79)	0.15 (0.07, 0.29)	0.31 (0.15, 0.59)	0.13 (0.06, 0.24)	0.19 (0.07, 0.38)	0.10 (0.04, 0.20)	1.19 (0.51, 2.54)	0.37 (0.17, 0.75)
**LAX-CA-Los Angeles**	0.17 (0.08, 0.32)	0.03 (0.01, 0.06)	0.13 (0.06, 0.25)	0.03 (0.01, 0.05)	0.09 (0.03, 0.18)	0.02 (0.01, 0.04)	0.45 (0.20, 0.94)	0.08 (0.03, 0.16)
**FLL-FL-Fort Lauderdale**	0.08 (0.02, 0.18)	0.04 (0.01, 0.09)	0.08 (0.02, 0.18)	0.04 (0.01, 0.08)	0.08 (0.02, 0.18)	0.04 (0.01, 0.08)	0.09 (0.03, 0.20)	0.04 (0.02, 0.09)
**BOS-MA-Boston**	0.10 (0.04, 0.20)	0.22 (0.07, 0.49)	0.09 (0.03, 0.18)	0.21 (0.06, 0.47)	0.07 (0.02, 0.16)	0.19 (0.05, 0.45)	0.20 (0.09, 0.39)	0.35 (0.15, 0.66)
**LAS-NV-Las Vegas**	0.09 (0.04, 0.19)	0.02 (0.01, 0.04)	0.07 (0.03, 0.14)	0.02 (0.01, 0.03)	0.03 (0.01, 0.07)	0.01 (0.00, 0.02)	0.30 (0.12, 0.68)	0.06 (0.02, 0.12)

Table S5. **Risk of YFV importation into US final destination airports from Southeast Brazil: counterfactual analysis, 2016-2017 outbreak.** Mean and 95% confidence interval of the estimated total number of YFV importations ($I_{S,A}^{W_S}$, comprising both Brazilian travelers and US and international travelers) entering the United States at the specified final destination airports during the 2016–2017 YF outbreak. Only estimates for Southeast Brazil at the regional and state level are included. ES = Espírito Santo, MG = Minas Gerais, RJ = Rio de Janeiro, SP = São Paulo, SE Brazil = Southeast Brazil.

5.2 2017–2018 YF outbreak

	Baseline		Sensitivity Analysis				Counterfactual Analysis	
	70% vaccination coverage travelers		No vaccination coverage of travelers		100% vaccination coverage travelers		No vaccination coverage in SE Brazil 2017-2018	
	SE Brazil (regional)	SE Brazil (state)	SE Brazil (regional)	SE Brazil (state)	SE Brazil (regional)	SE Brazil (state)	SE Brazil (regional)	SE Brazil (state)
**MIA-FL-Miami**	1.40 (0.66, 2.65)	1.08 (0.49, 2.13)	2.30 (1.00, 4.45)	1.52 (0.71, 2.85)	1.01 (0.43, 2.15)	0.88 (0.36, 1.92)	1.50 (0.71, 2.84)	1.20 (0.55, 2.39)
**JFK-NY-New York**	0.78 (0.36, 1.45)	0.54 (0.25, 1.03)	1.39 (0.57, 2.76)	0.81 (0.37, 1.54)	0.52 (0.22, 1.08)	0.42 (0.17, 0.89)	0.83 (0.40, 1.53)	0.60 (0.28, 1.18)
**MCO-FL-Orlando**	0.69 (0.32, 1.33)	0.50 (0.23, 1.02)	1.06 (0.48, 2.01)	0.66 (0.31, 1.26)	0.53 (0.22, 1.14)	0.43 (0.18, 0.94)	0.74 (0.35, 1.44)	0.57 (0.26, 1.17)
**ATL-GA-Atlanta**	0.33 (0.15, 0.60)	0.22 (0.10, 0.42)	0.58 (0.24, 1.16)	0.33 (0.15, 0.64)	0.21 (0.09, 0.45)	0.17 (0.07, 0.36)	0.34 (0.16, 0.63)	0.25 (0.12, 0.48)
**IAH-TX-Houston**	0.26 (0.12, 0.49)	0.18 (0.08, 0.35)	0.46 (0.19, 0.92)	0.27 (0.12, 0.51)	0.18 (0.08, 0.37)	0.14 (0.06, 0.31)	0.28 (0.13, 0.52)	0.21 (0.10, 0.40)
**FLL-FL-Fort Lauderdale**	0.21 (0.07, 0.46)	0.10 (0.04, 0.20)	0.56 (0.15, 1.28)	0.24 (0.07, 0.52)	0.06 (0.03, 0.13)	0.04 (0.02, 0.08)	0.22 (0.07, 0.46)	0.11 (0.04, 0.21)

Table S6. Risk of YFV importation into US first ports of entry from Southeast Brazil: baseline, sensitivity and counterfactual analyses, 2017-2018 outbreak. Mean and 95% confidence interval of the estimated total number of YFV importations ($I_{S,A}^{W_S}$, comprising both Brazilian travelers and US and international travelers) entering the United States at the specified first ports of entry during the 2017–2018 YF outbreak. Only estimates for Southeast Brazil at the regional and state level are included. SE Brazil = Southeast Brazil.

	Baseline		Sensitivity Analysis				Counterfactual Analysis	
	70% vaccination coverage travelers		No vaccination coverage of travelers		100% vaccination coverage travelers		No vaccination coverage in SE Brazil 2017-2018	
	SE Brazil (regional)	SE Brazil (state)	SE Brazil (regional)	SE Brazil (state)	SE Brazil (regional)	SE Brazil (state)	SE Brazil (regional)	SE Brazil (state)
**MIA-FL-Miami**	0.96, 0.45, 1.88)	0.72 (0.33, 1.48)	1.47 (0.65, 2.80)	0.94 (0.44, 1.80)	0.74 (0.30, 1.59)	0.63 (0.25, 1.38)	1.04 (0.48, 2.03)	0.81 (0.36, 1.70)
**JFK-NY-New York**	0.70 (0.33, 1.34)	0.49 (0.23, 0.97)	1.21 (0.49, 2.38)	0.73 (0.33, 1.38)	0.47 (0.20, 1.00)	0.39 (0.16, 0.84)	0.74 (0.35, 1.42)	0.55 (0.25, 1.10)
**MCO-FL-Orlando**	0.91 (0.42, 1.83)	0.70 (0.31, 1.46)	1.23 (0.57, 2.34)	0.84 (0.39, 1.65)	0.76 (0.31, 1.67)	0.63 (0.25, 1.40)	0.98 (0.44, 2.00)	0.80 (0.34, 1.70)
**LAX-CA-Los Angeles**	0.24 (0.11, 0.47)	0.17 (0.08, 0.35)	0.38 (0.17, 0.73)	0.23 (0.11, 0.44)	0.18 (0.07, 0.39)	0.14 (0.06, 0.32)	0.26 (0.12, 0.50)	0.20 (0.09, 0.40)
**FLL-FL-Fort Lauderdale**	0.19 (0.06, 0.41)	0.09 (0.04, 0.18)	0.48 (0.12, 1.10)	0.20 (0.06, 0.44)	0.06 (0.02, 0.12)	0.04 (0.01, 0.07)	0.19 (0.07, 0.41)	0.09 (0.04, 0.19)
**BOS-MA-Boston**	0.17 (0.08, 0.34)	0.13 (0.06, 0.24)	0.37 (0.12, 0.78)	0.24 (0.09, 0.49)	0.09 (0.04, 0.18)	0.07 (0.03, 0.15)	0.18 (0.08, 0.35)	0.13 (0.06, 0.25)

Table S7. **Risk of YFV importation into US final destination airports from Southeast Brazil: baseline, sensitivity and counterfactual analyses, 2017-2018 outbreak.** Mean and 95% confidence interval of the estimated total number of YFV importations ($I_{S,A}^{W_S}$, comprising both Brazilian travelers and US and international travelers) entering the United States at the specified final destination airports during the 2017–2018 YF outbreak. Only estimates for Southeast Brazil at the regional and state level are included. SE Brazil = Southeast Brazil.
